# Detecting non-allelic homologous recombination from high-throughput sequencing data

**DOI:** 10.1186/s13059-015-0633-1

**Published:** 2015-04-08

**Authors:** Matthew M Parks, Charles E Lawrence, Benjamin J Raphael

**Affiliations:** Division of Applied Mathematics, Brown University, Providence, USA; Center for Computational Molecular Biology, Brown University, Providence, USA; Department of Computer Science, Brown University, Providence, USA

## Abstract

Non-allelic homologous recombination (NAHR) is a common mechanism for generating genome rearrangements and is implicated in numerous genetic disorders, but its detection in high-throughput sequencing data poses a serious challenge. We present a probabilistic model of NAHR and demonstrate its ability to find NAHR in low-coverage sequencing data from 44 individuals. We identify NAHR-mediated deletions or duplications in 109 of 324 potential NAHR loci in at least one of the individuals. These calls segregate by ancestry, are more common in closely spaced repeats, often result in duplicated genes or pseudogenes, and affect highly studied genes such as GBA and CYP2E1.

**Electronic supplementary material:**

The online version of this article (doi:10.1186/s13059-015-0633-1) contains supplementary material, which is available to authorized users.

## Background

Non-allelic homologous recombination (NAHR) is a biological mechanism for repairing broken chromosomes, which results in gross genome rearrangements. A significant portion (approximately 10% to 22%) of all genome rearrangements in humans, also called structural variations, is thought to be the result of NAHR [1-5]. Understanding and detecting NAHR in individuals provide valuable insight for a wide variety of genomic disorders, disease susceptibilities, and cancers [6-14].

Despite its importance and prevalence, NAHR is challenging to detect with either computational or experimental techniques. The difficulty stems from four crucial properties: (1) NAHR is mediated by highly homologous repeats, (2) there are millions of repeats across the human genome [15], (3) the breakpoints of an NAHR-mediated rearrangement are always at homologous positions of homologous repeats, and (4) NAHR is capable of producing inversions, deletions, duplications, and translocations. Detection of NAHR thus requires a careful treatment of repetitive regions from the human genome. Currently, repetitive regions are a major weakness of biological and computational techniques for detection and verification[16]. Experimental techniques for discovering instances of structural variation, including array comparative genomic hybridization (aCGH), SNP microarrays, and fluorescence in situ hybridization (FISH), are frustrated by repetitive regions and thus encounter considerable difficulty in detecting NAHR [16]. While validations of NAHR have been done using these techniques [3,11,17], they are not used on nearly the same scale as high-throughput sequencing. New long-read sequencing technologies from Pacific Biosciences [18] and Oxford Nanopore [19] are providing a more comprehensive view of structural variation in the human genome [20,21], although the high single-nucleotide error rates and costs of these technologies have thus far limited their ability to detect NAHR events across many genomes.

High-throughput genome sequencing is a popular, powerful, and cost-efficient source of data for learning about genomes and inferring the variation between individuals. Typically, structural variations in an individual with respect to the reference are inferred from sequencing data by the mappings of paired-end reads to the reference genome. There are three main signals used to detect structural variation: discordant paired reads, split reads, and read depth [22]. Whenever the former two signals are used, it is always assumed that only the discordantly mapped or split reads indicate a structural variation,while the concordant reads were correctly mapped; this is true of BreakDancer, VariationHunter, GASV, PEMer, Pindel, HYDRA, CNVer, and GASV-pro^a^ [23-31]. But the biology of the NAHR mechanism requires that NAHR breakpoints occur at homologous positions of homologous repeats, making it highly likely that paired-end reads generated from NAHR breakpoints are mapped concordantly to the original repeats, albeit with a small number of mismatches. Thus, the very biology of NAHR implies that NAHR will very often go undetected by algorithms that rely on discordant or split reads, that is NAHR will be largely undetectable by most existing structural variation detection algorithms. Indeed, 93*%* of the NAHR breakpoints we find are supported by ≤2 discordant paired-end reads, meaning that these breakpoints are systematically ignored by most alignment-based algorithms. Also, many structural variation algorithms that utilize the read-depth signal, such as CNVnator and Event-wise Testing [32,33], are limited in their ability to detect copy number variants in repetitive regions due to mapping quality thresholds and selection of a mapping for reads with multiple possible alignments.

Directly modeling NAHR offers major advantages over generic predictions of structural variants from read data. The ‘rules of NAHR’ [11,34,35] have been studied and are well characterized [6-8,36], and provide a structured framework for detecting NAHR from short reads that is consistent with the biological mechanism. They determine where NAHR rearrangements may occur, what types of rearrangements are possible, and the exact location and sequence composition of the breakpoints. This has major implications for the analysis of sequencing data: the information provided by the rules of NAHR allows us to construct, fully and exactly, hypothetically rearranged genomes from which all reads were theoretically generated concordantly, thus bypassing entirely the notion of discordant read mappings. Further, the characteristics of NAHR and repeats indicate a natural way to evaluate read depth, freeing our model from relying on arbitrary bins and sliding windows. Thus, founding our model on the rules of NAHR provides a novel approach to high-throughput sequencing analysis for structuralvariation.

Data generated from repetitive regions require extremely careful analysis, since, by definition, there is very little signal to distinguish highly homologous repeats. Any computational model must be sufficiently sensitive to recognize such subtle differences in signal and, further, accumulate these differences to make inference informed by the entirety of the competing, often conflicting signals. Probabilistic models offer a natural way to capture such subtleties, and Bayesian models, in particular, use probability theory to weigh competing signals against each other to draw inference.

To capitalize on this, we developed a Bayesian algorithm that probabilistically models NAHR based on the rules of the mechanism and employs a specifically designed hidden Markov model alignment algorithm to probabilistically compare reads among repetitive sequences. We applied this model to potential NAHR events among low-copy repeats (LCRs) contained in a database of segmental duplications [15] and performed a Bayesian statistical inference on the occurrence of NAHR-mediated rearrangements in human genomes, focusing on deletions and duplications. Our model contains several key advances in the analysis of read data in the context of rearrangements due to NAHR, including a principled analysis of read depth inside repeats and the consideration of all possible mapping locations for every read. These features allow us to detect hitherto unreachable rearrangements in the human genome.

We analyzed publicly available, low-coverage Illumina paired-end sequencing data for 44 individuals from the 1000 Genomes Project using our model. Due to the repetitive nature of these regions and the limitations of experimental validation technology mentioned above [16], we restricted our calls to a reliable subset of 1,043 called NAHR events using a separate statistical test. Nearly all of our calls are novel when compared against several recent structural variation validation studies. Most of these called NAHR events are identified in only a subset of individuals (median of five individuals per locus with a call), and the called NAHR events show dependence of NAHR on ancestry, providing further evidence in support of the calls. We also assess the impact of NAHR on several highly studied genes, and we draw additional inference on general characteristics of NAHR.

This paper is organized as follows. First, we review the mechanism of NAHR in detail, highlighting characteristics that will be crucial to our mathematical model. Then, we apply a probabilistic framework to the mechanism and sketch the model. We then present the results of our model for a set of individuals, and discuss the biological significance of our results. An implementation of the algorithm described in this study, detect-NAHR, is freely available at [37].

### Mechanism of non-allelic homologous recombination

Here we briefly review the NAHR mechanism in humans. More detailed reviews can be found in [6,7]. Allelic homologous recombination (AHR) repairs double-stranded breaks (DSBs) in chromosomes by using the allele on the sister chromatid as a template. This mechanism is highly faithful because the allelic region of the sister chromatid is a nearly exact (up to polymorphism) copy of the DNA lost in the DSB. The crucial step in the AHR repair process is the homology search for locating the allele that is to serve as a template for repair of the DSB via PCR. If the DSB occurs in a unique region of the genome, then the homology search will almost certainly find the allelic position on the sister chromatid, and AHR will proceed as usual. But if the DSB occurs in or near a repeat, then the homology search may instead find a paralog of the repeat, making the ensuing repair non-allelic. If the ensuing double Holliday junction is resolved via crossover, then NAHR has occurred. The class of repeats that we examine for NAHR in this study are termed LCRs [38,39] or segmental duplications [15].

The type of rearrangement depends on the location of the paralog with respect to the chromatid with the DSB, and on the orientation of the two mediating repeats (positive orientation if the genomic index of both sequences increases along the alignment profile of the pair of repeats). Intra-chromatid NAHR results in deletions if the repeats are positively oriented, and inversions if negatively oriented. Inter-chromatid NAHR between positively oriented repeats results in deletions and duplications. Translocations result from positively oriented inter-chromosomal NAHR or negatively oriented repeats on different arms of the same chromosome.

The difficulty in detecting NAHR is that since the DSB region and the template for repair are highly homologous, the breakpoint region looks almost exactly the same before and after the rearrangement. Luckily, for any pair of repeats, there is often a small set of SNPs and short indels that distinguish the repeats; we refer to them as variational positions (VPs) (different from paralogous sequence variants; see Additional file 1: Section S7.22). Being conscious of the VPs, we see that since NAHR involves a crossover, then at the breakpoint the VPs switch from one repeat’s VP pattern to the other’s. Indeed, the repeat containing the breakpoint is actually a new repeat; it is a hybrid LCR, composed of part of each of the two repeats that mediated the NAHR rearrangement, joined at the breakpoint.

**Figure 1:**
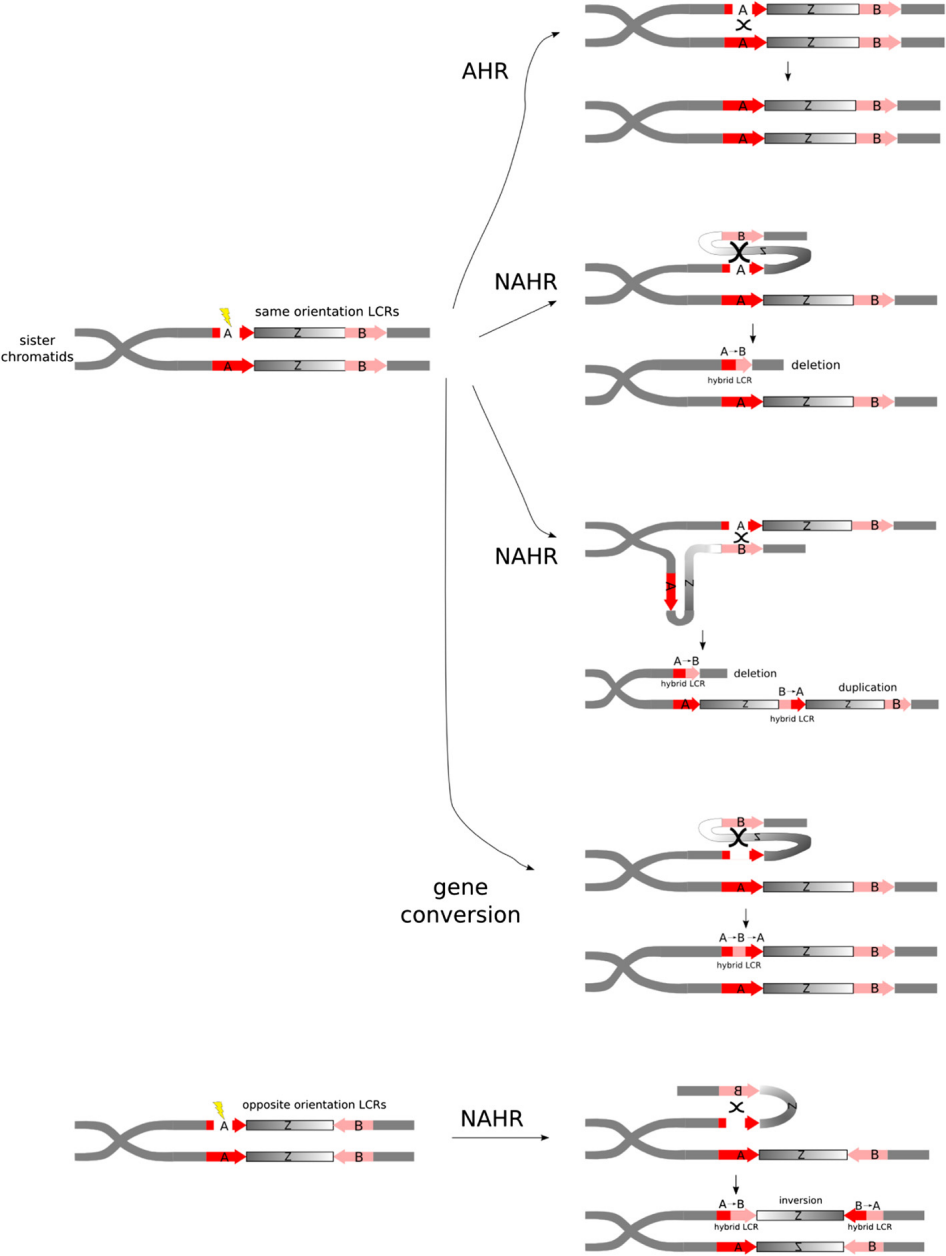
Schematic examples of homologous repair. Sister chromatids joined at the centromere are shown. Red and pink regions are homologous LCRs, labeled A and B, respectively. Arrow direction represents LCR orientation. The unique region between the homologous red and pink LCRs is labeled Z. The yellow bolt represents the location of a DSB in the read LCR. Several pathways for homologous repair are shown, with an intermediate stage in each case. In AHR, the red LCR is repaired using the allelic region on the sister chromatid (the other red LCR). The repaired sister chromatid is repaired perfectly, up to polymorphisms. NAHR occurs when a non-allelic but homologous region is used for a template to repair the DSB (that is one of the pink LCRs), and the double Holliday junction is resolved via crossover. This leads to the creation of a new LCR, which is composed of parts of each mediating LCR, called a hybrid LCR. This leads to deletions and/or duplications when the mediating LCRs have the same orientation (that is the arrows point in the same direction). When the LCRs have opposite orientation, NAHR results in an inversion. Finally, it may be that the double Holliday junctions formed during repair are resolved via non-crossover resolution, in which case a gene conversion event occurs. In the schematic example, the pink LCR donates a sequence to the red LCR via gene conversion. AHR, allelic homologous recombination; DSB, double-stranded break; LCR, low-copy repeat; NAHR, non-allelic homologous recombination.

We can characterize each NAHR rearrangement according to the VP pattern(s) exhibited in the resulting hybrid repeat(s). Of the two repeats that mediate an NAHR rearrangement, denote the one with smaller genomic indices as *A* and the other as *B*. Deletions result in a hybrid repeat with VP pattern *A*→*B*, with the original *A* and *B* repeats deleted. Duplications preserve both *A* and *B*, and additionally create a hybrid with VP pattern *B*→*A*. Inversions and translocations replace repeat *A* by a hybrid with pattern *A*→*B*, and replace repeat *B* by a hybrid with pattern *B*→*A*. Altogether, the resulting hybrid VP patterns and the types of rearrangements follow the rules of NAHR. The various outcomes described here are shown in Figure 1.

Lastly, it is important to mention the closely related gene conversion mechanism. Gene conversion follows exactly the same pathway as NAHR, but the double Holliday junctions are resolved via the non-crossover outcome. This does not produce a large-scale rearrangement as in NAHR; instead, it merely replaces a short tract of the genome by a copy of a donor tract. If *B* donates to *A*, then *A* is replaced by the hybrid *A*→*B*→*A*, and vice versa. See Figure 1. Notice that gene conversion events have two breakpoints, or rather, two switches in VP pattern. Although we do not focus on gene conversion in this study, it is necessary to include gene conversion in any model of NAHR since the breakpoint signals of a gene conversion event mimic those of NAHR events. For example, a gene conversion event producing the hybrid *A*→*B*→*A* could be easily mistaken as an NAHR deletion with hybrid *A*→*B* or an NAHR duplication with hybrid *B*→*A*.

## Results

We developed a model for detecting NAHR from paired-end read data that addresses many of the issues that typically arise due to repeats. Our model rigorously analyzes read depth inside repeats, termed repeat read depth, using a novel homology-based framework that is robust to read mappings inside repeats. The model also identifies NAHR breakpoints inside highly homologous repeats using hybrid reads – reads generated from the breakpoint of a new repeat formed from the hybridization of two other repeats – which hitherto have gone unnoticed and unstudied. The foundation of the model is based on the current biological knowledge of the mechanism of NAHR (the rules of NAHR [11,34,35]), and a database of segmental duplications [15], allowing our model to construct hypothetical NAHR breakpoint junctions exactly and align all relevant reads to them. Because any individual read will overlap a small number of VPs, many mapping algorithms often concordantly map reads generated from novel hybrid repeats to a specific existing repeat in the reference genome, resulting in what we term phantom concordance. Our model addresses this challenge by explicitly calculating the probability of each potential mapping location, including for candidate novel hybrid repeats, for every read mapped to a repeat in the reference. In so doing, it geometrically accumulates evidence from multiple reads for or against the existence of hybrid elements in an individual.

### Key features

Overall, explicitly modeling the outcomes of the NAHR mechanism allows our model to capitalize on several key features:

- We know exactly where NAHR events might occur and what their breakpoint regions would look like using a biological model of the mechanism of NAHR.
- We exactly construct hypothetical NAHR breakpoint junctions inside repeats (novel with respect to the reference) and manually align reads to them, allowing us to analyze hybrid reads that result from putative NAHR events and are distinguished by VPs.
- We evaluate all paired-end reads for evidence of NAHR breakpoints (in feature 2), not just discordantly mapped ones, preventing our model from overlooking hybrid reads that were mapped with phantom concordance to other areas of the genome.
- We evaluate read depth over groups of disjoint homologous regions, termed repeat read depth, rather than over consecutive linear subintervals of the genome (for example sliding windows or bins).

**Figure 2:**
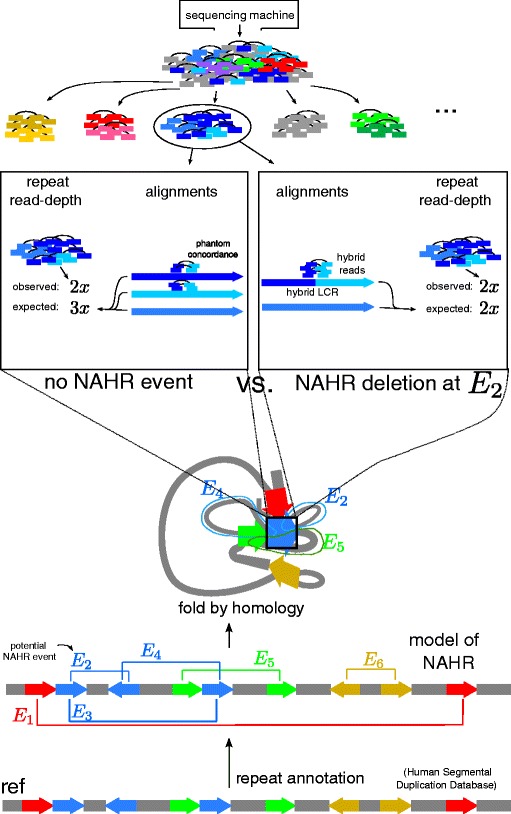
Schematic example of our model’s approach to detecting NAHR events from paired-end read data. The bottom half shows our framework for repeats and potential NAHR events. Each pair of homologous LCRs represents a potential NAHR event, annotated *E*_1_,…,*E*_6_. We then identify homologous LCRs into equivalence classes, folding the reference genome by homology. We focus on *E*_2_ and the blue LCRs for this example. We collect all paired-end reads homologous to the blue LCRs. In the middle, we analyze the schematic blue data for two cases: no NAHR event at *E*_2_ versus NAHR deletion at *E*_2_. For this schematic, assume that *E*_2_ indeed resulted in an NAHR deletion. According to the mechanism of NAHR, a deletion at *E*_2_ results in a hybrid LCR. There are two major components to each analysis: the repeat read depth and the alignment of reads. For the repeat read depth, we compare the observed number of reads across all blue LCRs against the expected number. In the no-event case, there are three blue LCRs and so we expect 3× blue reads, but we only observed 2× blue reads. For the NAHR deletion, a novel hybrid LCR was formed by hybridizing the dark blue and aqua blue LCRs; thus, we expect 2× blue reads, as observed. For alignments, we focus on the hybrid reads that span the NAHR breakpoint in the hybrid blue LCR. Since the hybrid blue LCR is novel with respect to the reference genome, then the hybrid reads can be mapped concordantly to blue LCRs in the reference with very few errors, which is termed phantom concordance. As they are mapped concordantly, they are ignored by most other existing structural variation detection algorithms. But when we align them against the hybrid LCR, many of the read errors are resolved. LCR, low-copy repeat; NAHR, non-allelic homologous recombination.

These concepts are summarized in Figure 2. We implemented our model in a C++ program that we call detect-NAHR. We describe each aspect of the model in detail below.

### Modeling non-allelic homologous recombination events and breakpoints

The foundation of NAHR is the pair of repeats that mediate the rearrangement. Any pair of highly homologous repeats may potentially mediate an NAHR rearrangement. For such a pair of homologous repeats, we refer to the interval on the reference genome from the beginning of the first LCR to the end of the second LCR as a potential NAHR locus, which may experience an NAHR event in the individual with respect to the reference.

We define the space of potential NAHR events as all possible pairs of homologous repeats in the human reference genome. The Human Segmental Duplication Database (HSDD) lists all pairs of sequences of length ≥1 kb and identity ≥90*%*, termed segmental duplications or LCRs [15,39] (obtained from [40]), on the reference (GRCh37). We consider the space of repeats to be the LCRs listed in the HSDD (see Additional file 1: Section S7.24 for justification).

Thus, every entry in the HSDD (that is a pair of homologous LCRs) therefore represents a distinct potential NAHR event *E*, where *E* is a random variable whose potential NAHR outcomes are determined by the locations and orientations of its pair of LCRs; that is the rules of NAHR as in Section ‘Mechanism of non-allelic homologous recombination’. In this study, we restrict our attention to deletion events and duplication events.

The relationship between the potential NAHR events *E*_1_,…,*E*_*n*_ is complicated by the layout of LCRs across the genome. For example, between a pair of homologous LCRs might lie a third LCR homologous to neither of its flanking neighbors. Therefore, when calculating the probabilities of various NAHR scenarios, events at certain potential NAHR loci must be considered simultaneously because an event at one locus may have implications for events at another locus. For illustration, Additional file 1: Figure S12a) contains a schematic genome and several hypothetical NAHR events. Therein, we see that potential NAHR events *E*_3_ and *E*_4_, although mediated by non-homologous LCRs, do in fact impact each other: if *E*_3_ occurs as an NAHR deletion, then one of the mediating LCRs for *E*_4_ is deleted, and hence *E*_4_ cannot undergo NAHR, and vice versa. This is an exclusivity constraint between *E*_3_ and *E*_4_ (see [41] for an analysis of exclusivity constraints). In addition, *E*_4_ and *E*_6_ must be considered simultaneously because the mediating LCRs are all homologous. On the other hand, *E*_4_ and *E*_5_ are not related because their mediating LCRs are not homologous and the regions potentially affected are non-homologous and disjoint. All of the types of relationships illustrated here are unambiguous and completely determined by the layout of the reference genome. Thus, potential NAHR events in the reference genome have varying degrees of complexity, that is the number of other potential NAHR events whose outcomes must be considered simultaneously. Note that this extends beyond the notion of repeat families: potential NAHR events are related not only because the mediating LCRs share homology, but because the intervening regions may intersect or be partially homologous.

When an NAHR event *E* occurs, it must have a breakpoint *B*. We assume that *B* occurs somewhere within the two LCRs. Technically, the breakpoint can be at any position along the LCRs. But given a pair of consecutive VPs on a repeat, all possible breakpoints occurring in the interim region will therefore result in exactly the same hybrid repeat. We therefore restrict the space of potential NAHR breakpoints to be the set of VPs that distinguish a given pair of repeats.

VPs have been investigated and utilized in NAHR detection previously [3,11,42,43], although not explicitly named as such. For example, Ou et al. used VPs to locate the breakpoint region of experimentally validated NAHR events in the same way we describe above [11], showing the switch in VP pattern surrounding the breakpoint region.

The rules of NAHR thus provide us with a well-defined space of possible outcomes and breakpoints. For any putative rearrangement, we may therefore exactly construct the entire affected region, including the breakpoint within the LCRs. Being able to construct every hypothetical outcome and calculate its probability, we compare all possibilities against each other via Bayes’ rule (see ‘Materials and methods’).

### Read depth

We introduce a novel approach for evaluating read depth, called repeat read depth, which considers read depth over collections of homologous regions. Since we group homologous regions together, we avoid the classic issue of uncertainty in the mappings of short reads into repeats. Indeed, for evaluating read depth across homologous LCRs, we do not need to worry which paralog a certain read came from, but only that it came from some paralog of an LCR. Thus, we never attempt to determine the correct mapping for any read at any stage in our model.

Instead, we address repetitive regions by associating homologous regions of the genome with each other, that is forming equivalence classes of homologous regions, in a manner similar to the de Bruijn and A-Bruijn graph formulations by Pevzner [44,45] (see Additional file 1: Section S7.12). Knowing exactly which regions compose each equivalence class, we use probability theory to determine the impact of a given NAHR event and breakpoint on the expected read depth of a certain set of regions. We model the number of reads in a region as a negative binomial distribution (an overdispersed Poisson),approximated by a normal in large regions. This form is based on studies that have found that the distribution of fragments along the reference genome has greater variation than a Poisson distribution [46], and is biased by the GC content of the fragment [46,47].

### Read alignments

**Figure 3:**
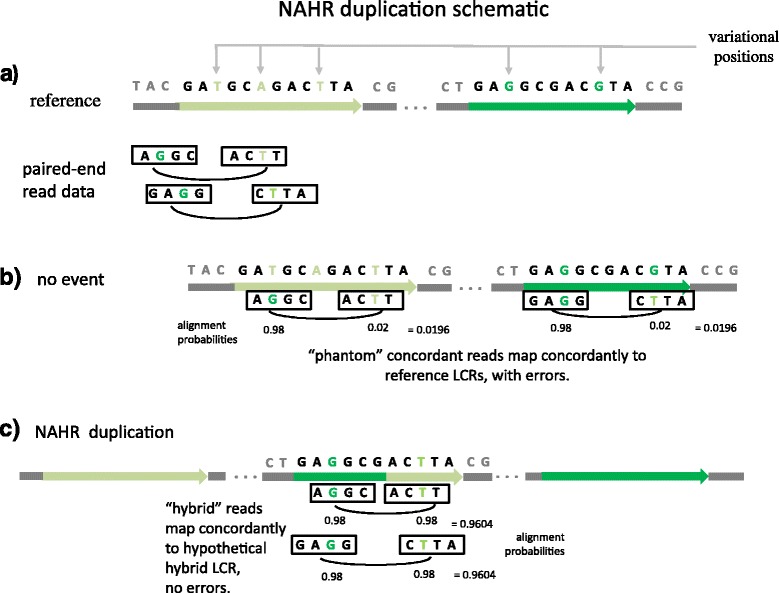
Schematic example of an NAHR duplication with paired-end read data.**(a)** A schematic reference genome and paired-end read data. The dark green and light green regions are homologous LCRs that form a potential NAHR event locus. Green nucleotides are the VPs that distinguish the two LCRs. We suppose the individual experienced an NAHR duplication at this locus, and that the two paired-end reads shown were generated from the breakpoint region, that is they are hybrid reads. We consider two possible outcomes: no event and an NAHR duplication. **(b)** If no NAHR event occurs at this locus, then this locus of the individual’s genome is the same as in the reference. Notice that the paired-end reads are aligned concordantly to these LCRs, albeit with a small number of errors at the VPs; we call this phantom concordance. Suppose the probability of a read error is 2*%*. Then here, the likelihood of the mediating LCR is 0.98×0.02=0.0196 for each paired-end read. **(c)** The hybrid LCR formed from the NAHR duplication event is shown with aligned paired-end reads. The hybrid LCR is novel to the individual; it does not exist in the original reference genome. Notice that the VPs switch from dark green to light green after the breakpoint in the hybrid LCR. For simplicity in this schematic, we calculate the probability of a paired-end read’s alignment according to the agreement between its mates’ bases at the VPs, although in the algorithm a full alignment probability is calculated. The likelihood that the paired-end reads came from the hybrid LCR is 0.96^2^=0.9604 for each read. LCR, low-copy repeat; NAHR, non-allelic homologous recombination; VP, variational position.

We do not take any read mappings as given. Instead, given a collection of possible NAHR events, we consider all of the locations in the individual’s hypothetical genome that may have (concordantly) generated each paired-end read. Paired-end reads that were generated from an NAHR breakpoint, that is hybrid reads, can be mapped to either of the two mediating LCRs (*A* or *B*) with few mismatches, that is with phantom concordance. Figure 3 shows a schematic example of paired-end reads generated from the breakpoint region of an NAHR duplication. Such paired-end reads may contain the switch in VP pattern, and thus provide evidence completely analogous to the evidence presented by Ou et al. [11] and Kidd et al. [3] when justifying breakpoint calls in repetitiveregions.

Because multiple independent reads increase the probability of a breakpoint in proportion to the product of the probabilities of the individual reads, even a limited number of supporting reads can provide strong evidence of a breakpoint. Figure 3 gives a schematic illustration of the power gained from two informative reads, each containing only two VPs.

To calculate the probability of a set of reads given an individual’s hypothetical genome, we consider eachpossible generating location to be *a priori* equally likely and calculate each read’s likelihood of each generating location using the context-sensitive hidden Markov model (Additional file 1: Section S7.14). For reads withhomology to the regions surrounding NAHR breakpoints in the hypothetical genome, we construct the NAHR breakpoint junction (that is, a novel sequence with respect to thereference) and perform pairwise alignments of all relevant paired-end reads to the NAHR breakpoint region using the context-sensitive conditional hidden Markov model; for more details, see Additional file 1: Section S7.21.

### Simulations

To estimate the sensitivity and specificity of our model, we randomly drew 20 one-copy NAHR events, changed the reference genome accordingly, simulated 15× paired-end read data, and tested our model’s ability to recover the NAHR events (details in Additional file 1: Section S7.20). The 20 drawn NAHR events served as positive controls, while the remaining 304 potential NAHR loci without a drawn event served as negative controls. We repeated this procedure 18 times. Our estimated specificity and sensitivity are 99.8*%* and 61.4*%*, respectively, indicating that our model is conservative and does not make a large number of false positivepredictions.

### Non-allelic homologous recombination calls on real data

As discussed above, both experimental validation and computational analysis of NAHR is extremely difficult due to the repetitiveness of the relevant regions [16]. To address the issues of validation in repeat regions, we developed a set of statistical tests for NAHR regions to be used to isolate a conservative set of reliable NAHR event calls.

#### False discovery rate via read depth

Our model integrates read depth and paired-end read alignments from both unique and repetitive regions into a single, joint probabilistic model of high-throughput sequencing data and NAHR events with breakpoints. An NAHR deletion or duplication call can be evaluated by a simpler statistical test that is separate from our Bayesian model and uses only the read depth in the putatively affected region.

The statistic we chose for the read depth of a region is the ratio *γ* of the observed number of reads over the expected number of reads under the null hypothesis (no NAHR event). The expected number of reads is sensitive to GC, following Speed & Benjamini [46]. Note that *γ* does not depend on the length of the region at hand. If there was no event, we would expect *γ*≈1. We calculated the false discovery rate (fdr) for each potential NAHR locus from *γ* in a manner based on the method developed by Efron [48]. Details of this test are in Additional file 1: Section 7.17.

#### Breakpoint log odds

Modeling the mechanics of NAHR allowed our model to construct every possible breakpoint region and to align reads against them. We quantify the evidence of a called breakpoint by calculating an odds ratio of alignment probabilities for reads relevant to the breakpoint region. Given a breakpoint *B*, we may denote a small region around *B* and all paralogous regions as , and collect all reads mapped to a region in . The likelihood *P*_0_ of the null hypothesis (there was no NAHR, and so *B* is not a breakpoint) can then be computed by aligning each read to every location in . The likelihood *P*_*A*_ of the alternative hypothesis (*B* is indeed the breakpoint) is calculated by including the newly formed hybrid breakpoint region in , removing from  the pair of regions that together form the hybrid, and aligning each read to each region in this modified set. The log-odds ratio log*P*_*A*_/*P*_0_ represents how much more likely the existence of a specific breakpoint is compared to the null case (no breakpoint).

### Conservative call set

We analyzed low-coverage Illumina paired-end read data for 44 individuals obtained from the publicly available database of the 1000 Genomes Project, focusing on the detection of NAHR deletions and duplications. We chose the 44 low-coverage individuals with the largest datasets.^b^ We analyzed the same 324 potential NAHR deletion/duplication loci for each individual. This subset of all possible NAHR loci was chosen strictly based on computational constraints (following Section ‘Modeling non-allelic homologous recombination events and breakpoints’, we chose the 324 loci with the smallest computational complexity, as determined by the number of potential NAHR loci that must be simultaneously considered during probability calculations). Analyzing the same 324 potential NAHR events across 44 individuals gives a total space of 44×324=14,256 possible NAHR event calls. To isolate a reliable set of NAHR deletion/duplication calls, we required a candidate call to have fdr ≤0.01 according to the repeat-sensitive read-depth test of Section ‘False discovery rate via read depth’. We found that this fdr threshold gives a good separation between positive and negative NAHR calls (Additional file 1: Section S7.7 and Additional file 1: Figures S9 and S10).

Our results are summarized in Table 1. Of our NAHR event calls, 1,043 passed the fdr threshold, which were called at 109 distinct potential NAHR loci when collapsed across the 44 genomes. Of the 1,043 calls, 722 were duplications and 321 were deletions. Notice that the total number of distinct loci with a positive NAHR event call in some individual is not the sum of the number of such distinct loci for deletions and duplications separately; this is because some loci were called as NAHR deletions in some individuals, but were called as NAHR duplications in others. The median number of positive NAHR calls per individual is 24 (7.41% of all 324 tested loci). Comparing against structural variation calls and experimentally validated rearrangements reported in [3,4,17], we found that only 106 of our 1,043 calls (21 of the 109 distinct loci with a positive NAHR event call) were previously reported, and of the 106 previously reported calls, 59 were positively experimentally validated (see Additional file 1: Section S7.6).

**Table 1:** **Summary statistics**

	**Number of loci**	**Number of distinct**	**Median calls/**	**Median number**	**Number previously**	**Number positively**	**Median affected**	**Number in**	**Number in**	**Number in**
	**with an event call** ^**a**^	**loci with an NAHR**	**person** ^**b**^	**of people/locus**	**reported** ^**c**^	**validated** ^**d**^	**genes/person**	**Africans** ^**c**^	**Asians** ^**c**^	**Europeans** ^**c**^
		**event call** ^**b**^		**with call**						
Deletion	321 (2.25%)	65 (20.1%)	5.5 (1.7%)	4 (9.09%)	106 (33.0%)	59 (18.4%)	7.5	120 (37.4%)	123 (38.3%)	78 (24.3%)
Duplication	722 (5.06%)	64 (19.8%)	16 (4.94%)	3 (6.82%)	0	0	42.5	311 (43.1%)	186 (25.8%)	225 (31.2%)
Total	1,043 (7.32%)	109 (33.6%)	24 (7.41%)	5 (11.4%)	106 (10.2%)	59 (5.7%)	52	431 (41.3%)	309 (29.6%)	303 (29.1%)

^a^Out of all 44×324=14,256 potential NAHR event loci.

^b^Out of all possible 324 distinct loci.

^c^Out of the number of positive event calls.

^d^Subset of number previously reported. Validations from [3,4,17]. Non-positively validated calls were not negatively validated; see Additional file 1: Section S7.6.

Since our model evaluates both read depth and read alignments to make NAHR event calls, it is possible for our model to make a high-confidence NAHR event call at a locus due to a strong read-depth signal, and yet have much lower confidence about the location of the breakpoint of that NAHR event. We can identify the subset of our positive NAHR event calls that have strong evidence of a breakpoint by imposing a threshold of 6 on the breakpoint log odds (see Section ‘Breakpoint log odds’) for each called NAHR event (see Additional file 1: Section S7.25 for the choice of threshold). Of the 1,043 positive NAHR event calls across the 44 individuals, 512 calls had breakpoints with log odds ≥6. Below, we analyze in detail the impact of our positive NAHR calls on genes using the 512 NAHR calls with high-confidence breakpoints.

To understand the kinds of reads that supported our 512 high-confidence NAHR breakpoint calls, Additional file 1: Figure S7 contains a histogram of the number of discordant paired-end reads supporting each such call. Recall that nearly all other structural variation algorithms detect structural variation using only discordantly mapped reads. But of the 512 NAHR event calls with a high-confidence breakpoint, 425 (83*%*) were supported by zero discordant paired-end reads, guaranteeing them to be undetectable by other algorithms. Another 10.4*%* would be very unlikely to be detected by other algorithms since so few (≤2) discordant reads support them. Thus, most of the support for our high-confidence NAHR breakpoints comes from paired-end reads that were mapped with phantom concordance, that is mapped concordantly to a highly homologous region of an LCR from which they were not actually generated. Note that 90*%* of the 512 high-confidence breakpoints were supported by ≥4 hybrid reads (see Additional file 1: Section S7.5).

### Non-allelic homologous recombination events across individuals and relation to ancestry

Repetitive regions pose difficulties not only for detecting rearrangements, but in constructing the reference genome as well. As such, an immediate concern would be that putative NAHR rearrangements reflect anomalies in the reference genome rather than genuine rearrangements. In such cases, we would expect that all (or nearly all) of the individuals tested would display such a signal. Further, the erroneous signal displayed by each individual would perhaps be of slightly different magnitude, and a criticism could be that our model merely chooses some of the individuals to make a call on according to some arbitrary threshold imposed on a signal that does not actually separate the data well.

Additional file 1: Figure S9 shows that, in general, a given potential NAHR locus has NAHR event calls in only a subset of the 44 tested individuals. Among the loci in which an NAHR event was called positive in at least one individual, the number of individuals with some NAHR event at a particular locus has median 5 (11.4%), which is far from all 44. Further, only 7 (6.4%) loci had a positive NAHR event call in 34 (77.3%) of the tested individuals.

Additionally, if our called NAHR events are true genetic polymorphisms (as opposed to artifacts), then their presence or absence among different individuals should be correlated with ancestry. To explore the relationship of our detected NAHR events to ancestry, we tested the hypothesis that the occurrence of NAHR events was independent of ancestry. Of our 1,043 calls passing the fdr threshold, 431 calls (41.3%) were in individuals of African ancestry, 309 (29.6%) in individuals of Asian ancestry, and 303 (29.1%) in individuals of European ancestry. These numbers are reasonable, as the reference genome is European. Testing the relationship between ancestry (African, Asian or European) and NAHR loci (109 distinct loci with a positive NAHR event call), we find that ancestry and NAHR events are overall not independent (*χ*^2^=302.8, degrees of freedom =216 and *P*=8.8×10^−5^). That is, the occurrence of NAHR deletions and duplications across the genome is not independent of ancestry. Because of the limited sample size (44 individuals), we were unable to identify specific ancestry-related events that are statistically significant after correction for multiple comparisons.

### Impact on genes

We searched a database of Ensembl genes on the reference genome (obtained from BioMart [49]) and found that 216 genes were affected^c^ by the 109 distinct loci with some NAHR event call passing the fdr threshold. The median number of affected genes per individual was 52. The affected genes included several highly studied genes, such as hemoglobin (HBA1, HBA2, HBMA and HBZ), haptoglobin (HP and HPR), and those involved in drug metabolism (CYP2E1). A full list of genes affected by our called NAHR events can be found in Additional file 1: Section S7.2.

A more detailed analysis of the impact of an NAHR event on a gene depends on the relative locations of the gene, the mediating LCRs, the NAHR breakpoints, and any pseudogenes. The simplest case is when a gene is contained in the region between the two mediating LCRs, but not intersecting either LCR; then an NAHR deletion or duplication will completely delete or duplicate the gene, respectively. If a gene is contained within one of the mediating LCRs then the exact locations of the breakpoints are very important. If the gene lies within the breakpoints, then it will be completely deleted or duplicated, as before. If the gene lies outside of the breakpoints, then it will be physically unaffected, although the distance to its promoter or regulatory elements may change. If the breakpoint intersects the gene, then a new fusion gene will arise. The composition of this fusion gene will depend on what was lying at the homologous position on the other LCR: another gene or a pseudogene. Finally, sometimes a gene actually contains a pair of LCRs (as does the haptoglobin gene *HP*, for example), in which case, an NAHR deletion or duplication will cause a contraction or expansion of the gene, respectively. Clearly the exact location of the breakpoint within the gene will have major implications for transcription. Several instances of these scenarios are highlighted below in Section ‘Case study’ and Additional file 1: Table S6.

NAHR is an important evolutionary mechanism for the creation of pseudogenes and the creation of novel genes via fusion or contraction/expansion. For each of our 512 NAHR event calls with a high-confidence breakpoint (see Section ‘Conservative call set’), we searched a database obtained from BioMart[49] of all Ensembl genes and pseudogenes to determine the impact of the called NAHR event and breakpoint. Table 2 contains the results. In particular, notice that 381 genes and 12 pseudogenes genes were duplicated completely, and 19 novel genes were formed via fusion.

**Table 2:** **Breakpoint impact on genes**

**Impact**	**Number of NAHR calls**
Fusions: gene–gene	16
Fusions: gene–pseudogene	3
Fusions: pseudogene–pseudogene	1
Deleted genes	39
Deleted pseudogenes	14
Duplicated genes	381
Duplicated pseudogenes	12
Expanded genes	251
Expanded pseudogenes	46
Contracted genes	52
Contracted pseudogenes	3
Unaffected genes on LCRs	403
Unaffected pseudogenes on LCRs	10

Of our 1,043 positive NAHR calls across the 44 individuals, 512 had highly confident breakpoints (log-odds ratio ≥6). Checking these 512 breakpoints against databases of genes and pseudogenes, we determined the impact of each breakpoint on various genes and pseudogenes. Note that one NAHR event may affect multiple genes (see Additional file 1: Table S4 and Figure S11 for examples), and so the total number of genes affected may not be equal to the number of called breakpoints passing the log-odds ratio threshold. NAHR, non-allelic homologous recombination.

### Case study

We now demonstrate the various facets of our model by investigating a single NAHR event call in detail: a two-copy duplication of 20.5 kb on chromosome 1 with GRCh37 reference breakpoints 155,184,704 and 155,205,331 for Yoruban individual NA19129. This rearrangement is novel; it was not reported in any of the previous validation studies [3,4,17]. The called breakpoints are deep inside the mediating LCRs: 4,531 bp and 4,564 bp inside LCRs of lengths 10,583 and 12,491, respectively. This highlights the crucial role that VPs play in detecting breakpoints of NAHR events inside repeats, as we describe in detail below. Indeed, the called breakpoints are nearly right in the middle of the mediating LCRs, far away from any flanking unique regions that could have been used to anchor mates of any overlapping paired-end reads, as some algorithms attempt to do.

Note that we detected an NAHR event at this locus in exactly one other individual: NA19190, also Yoruban. The call for NA19190 was identical to that for NA19129: also a two-copy duplication, and with the same breakpoints.

#### Hybrid reads

**Figure 4:**
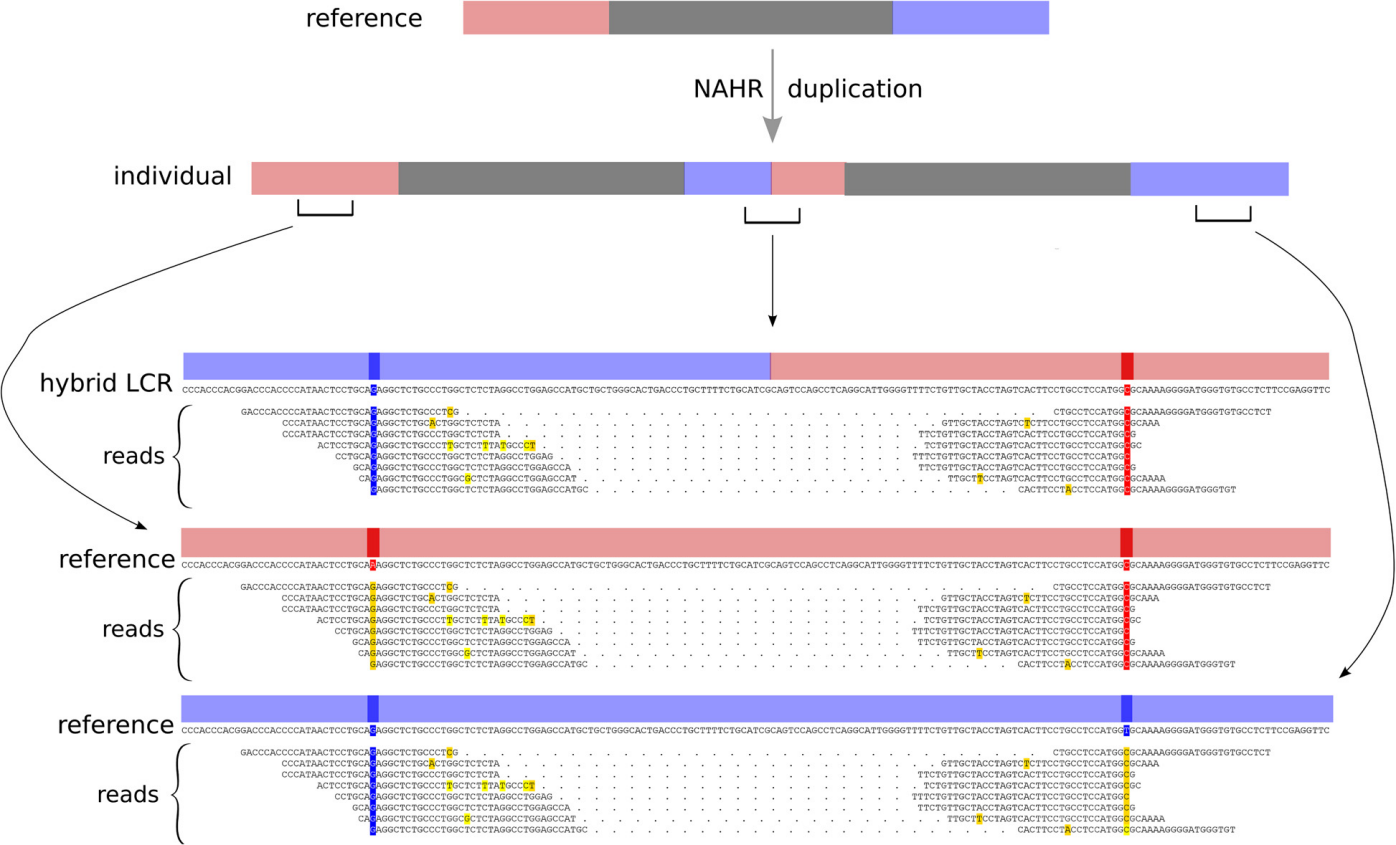
Multiple alignments of the paired-end reads that display the expected switch in VP patterns. These were at the breakpoint of a duplication on chromosome 1 with breakpoints 155,184,704 and 155,205,331 for Yoruban individual NA19129. The called NAHR duplication was mediated by LCR *A* (red) with coordinates [155180173,155190755] and LCR *B* (blue) with coordinates [155200767,155213257]. At the top is a schematic representation of the reference genome (not to scale) in the region chr 1:[155180173,155213257]. For presentation, we collected all paired-end reads that display the expected switch in VPs. This same collection of paired-end reads (mates connected by dots) are shown in three multiple alignments: against the predicted hybrid LCR; against LCR *A*; and against LCR *B*. VPs are colored according to which reference LCR’s VP pattern they agree with. Positions in the reads that disagree with the reference/hybrid LCR are colored yellow, while those that agree are colored appropriately. When aligning the reads to LCR *A*, notice that the reads perfectly agree at the VPs on the right-hand side of the alignment, but completely disagree with the VPs of the left-hand side of the alignment. But when aligning the reads to LCR *B*, the situation is reversed. Finally, when aligning to the hybrid LCR, all disagreements between the reads and the reference are resolved. The log-odds ratio of the probability of the reads given that there was no NAHR event (that is the hybrid does not exist) versus the probability of the reads given that the two-copy duplication indeed occurred (that is the hybrid LCR does exist) is −13; strong support for the two-copy duplication and the specific hybrid LCR. LCR, low-copy repeat; NAHR, non-allelic homologous recombination; VP, variational position.

For illustrative purposes, we collected all paired-end reads for NA19129 that displayed the switch in VPs as implied by the called NAHR two-copy duplication breakpoints. Figure 4 shows a multiple alignment of these paired-end reads against each of the LCRs *A* and *B* that mediated the NAHR duplications, and against the hybrid LCR *BA* resulting from these duplications. There are two VPs *v*_1_ and *v*_2_ of interest at this locus of the mediating LCRs.^d^ Our model called a two-copy NAHR duplication between LCRs *A* and *B*, with both duplications having a breakpoint in the region [*v*_1_+1,*v*_2_].^e^ When the reads are aligned against LCR *A*, then all of the reads agree with the reference at *v*_2_, but disagree with the reference at *v*_1_, and they all display the same incorrect base (G instead of A). On the other hand, when the reads are aligned against LCR *B*, the situation is reversed. Finally, when the reads are aligned to hybrid *BA*, which would hypothetically result from an NAHR duplication with breakpoints in the region [*v*_1_+1,*v*_2_], then all of the reads agree with the reference at both *v*_1_ and *v*_2_. This is very strong evidence in favor of the hybrid over either mediating LCR; indeed, the log-odds ratio (see Section ‘Breakpoint log odds’) of the reads aligning to the mediating LCRs versus the hybrid LCR is 12.3.

Unaware of the rules of NAHR, a naive approach may dismiss the disagreements at *v*_1_ and *v*_2_ as SNPs or read errors, or may ignore the reads for containing too little information. For example, of the eight paired-end reads shown spanning the breakpoint, all of them were mapped concordantly by BWA (and designated as properly paired) [50]. Further, seven of the eight paired-end reads have a mate with mapping quality 0. Mates that are given mapping quality 0 are considered to contain too little information to be confidently mapped to a unique location, and are ignored by many structural variation algorithms, including BreakDancer and PEMer [23,27]. But when we precisely construct the hypothetical hybrid LCR that results from an NAHR duplication at this locus according to the rules of NAHR, we see that in fact both mates of all of these paired-end reads contribute a significant amount of power: seven out of eight of the reads have a posterior probability >0.96 for mapping to the hybrid LCR as opposed to either mediating LCR. Thus, although the difference in signal between the hybrid LCR and the mediating LCRs is slight (literally a few SNPs), there is much discriminative power to be gained from the low read-error rate (approximately 2%) and from multiple reads displaying the signal.

For contrast, we considered all paired-end reads homologous to the region about the same called breakpoints, but in European individual NA07051 and Yoruban individual NA18501, for whom we did not call any NAHR events at this locus. To summarize the above, for NA19129, we found eight paired-end reads informative of the called breakpoint that displayed a switch in VP; that is, eight reads simultaneously correctly matched VPs from both mediating LCRs. On the other hand, for NA07051 there were eight paired-end reads potentially informative of a breakpoint at the same location as called for NA19129. However, zero of them correctly matched VPs from both LCRs, that is displayed a switch in VPs consistent with a hybrid LCR. Instead, five paired-end reads correctly matched VPs from the first mediating LCR but not the second, while three paired-end reads correctly matched the second mediating LCR but not the first. The situation is similar for NA18501. The corresponding multiple alignments for NA07051 and NA18501 of all paired-end reads in the region of the breakpoint called for NA19129 are in Additional file 1: Section S7.10.

#### Read depth

**Figure 5:**
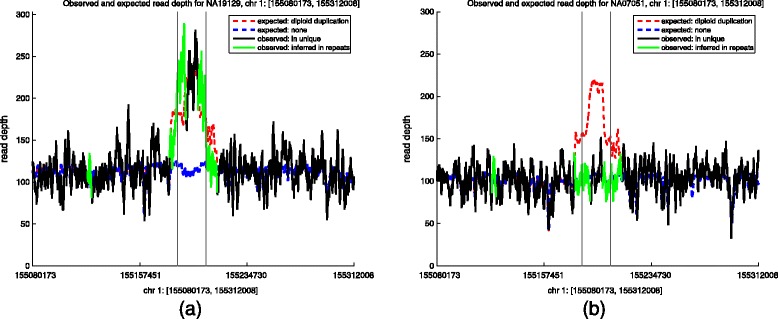
Observed and expected read-depth signals for a two-copy duplication on chromosome 1. There are breakpoints 155,184,704 and 155,205,331 for two individuals. The mediating LCRs have coordinates [155180173,155190755] and [155200767,155213257]. For perspective, the observed and expected read depths are shown for an additional 100 kb flanking the breakpoints. The expected read-depth signal appears as a dotted line; red for the called NAHR two-copy duplication, blue for no NAHR event. The solid green line is the inferred observed read-depth signal in repetitive regions (see Section ‘False discovery rate via read depth’). The solid black line is the observed read-depth signal in the unique region of this locus. Vertical thin black lines mark the called breakpoints. Read-depth curves are calculated as sliding 1,250-bp window sums for presentation. The expected read-depth signals are highly non-uniform due to the GC-bias in fragment distribution (see Section ‘Read depth’). **(a)** Read depth in unique and repeat regions for NA19129. Notice that the observed read-depth signal for the unique sequence in between the mediating LCRs follows the expected read-depth signal of a two-copy NAHR duplication (red dotted line) much closer than the expected read-depth signal if there was no NAHR event (blue dotted line). We also see the inferred observed read-depth signal transition from closely following the expected no-event signal (blue) to the expected duplication signal (red) and back again at approximately the location of the breakpoints. Together, the observed read-depth signal in the unique regions and the inferred observed read-depth signal in the repetitive regions give an fdr =1.3×10^−3^; strong support for the proposed two-copy NAHR duplication. **(b)** Unique read depth and inferred repeat read depth is shown at the same locus for European individual NA07051. Our model determined there was not an NAHR event for this individual at this locus. The fdr at this locus for NA07051 is >0.99. chr, chromosome.

In Section ‘False discovery rate via read depth’, we infer the read depth in the repetitive regions and plot its signal in Figure 5a alongside the unique read-depth signal. Note that the signal in repetitive regions transitions from following the expected signal for no event to the expected signal under the called two-copy duplication, as we expect. Further, the transition from null to alternative expected read depth occurs near the called breakpoints; this shows the significant amount of information contained even in reads from highly repetitive regions. Including inferred read depth from repeat regions together with the unique region, we calculate the fdr to be 1.3×10^−3^. This again indicates that a two-copy duplication is much more likely than a null event at this locus. Indeed, we were able to detect NAHR events involving only repetitive regions (for example NAHR between tandem LCRs), as shown in Additional file 1: Table S6.

For perspective, Figure 5b shows the unique and inferred read-depth signal at the same locus for European individual NA07051. Our model determined that this individual did not have an NAHR event at this locus. The fdr for this locus for NA07051 individual is >0.99. It is already obvious from the read-depth signal graph alone that, indeed, individual NA07051 did not experience an NAHR event at this locus, and the fdr reinforces this conclusion. This also serves as another particular example of the strong difference in signal between positively called NAHR events and negatively called ones.

#### Relations to disease

The two-copy NAHR duplication studied here affects the genes *GBA* and *MTX*1 and their respective pseudogenes *GBAP*1 and *MTX*1*P*1. Mutations in *GBA* cause Gaucher’s disease and are strongly associated with Parkinson’s disease in populations worldwide [51,52]. Mutations in *MTX*1 have also been linked to Parkinson’s disease [52]. Somatic mutations in *GBA* have also been linked to lung cancer, and somatic mutations in *GBAP* and *MTX*1 have also been linked to endometrium cancer [53].

The gene context of our two-copy NAHR duplication is shown in Additional file 1: Figure S11. According to our called breakpoints, two identical fusion *GBA* genes are created from the called two-copy NAHR duplication. The fusion genes consist of the first 1.1 kb of *GBA* followed by the last 12.5 kb of pseudogene *GBAP*1. The breakpoint occurs 1,093 bp inside GBA, and is 901 bp inside the coding region of GBA. Two additional complete copies of the pseudogene *MTX*1*P*1 are alsoformed.

### Other important examples of non-allelic homologous recombination

To highlight the biological impact of NAHR, we briefly present four more positive NAHR event calls and their impact on several highly studied genes, including RNASE2, RNASE3, FLG, CYP2E1, SPRN, SYCE1, HP, HPR, and TXNL4B. Additional file 1: Table S6 contains basic information for each called NAHR event, as well as figures similar to those above demonstrating the read-depth signal, and genome context. This table also describes the genes affected by each call and their functions or associated genomic disorders. All calls presented in the table are novel.

## Discussion

We have developed a Bayesian probabilistic model for detecting NAHR using high-throughput sequencing data. To our knowledge, our model is the first to utilize the specific features of the molecular mechanisms involved in NAHR. We also modeled the generation of high-throughput sequencing data, including biases in fragment distribution and error-rates during base generation as presented in recent literature.

To obtain a set of highly reliable NAHR event calls, we applied a read-depth-based fdr analysis to our initial calls and retained only those with fdr ≤0.01. The result is a set of 1,043 highly reliable calls across the 44 tested genomes, composed of 321 deletions and 722 duplications (see Additional file 1: Section S7.27). Collapsing these 1,043 across individuals, we arrive at a set of 109 distinct NAHR loci with a positive NAHR event call. We selected one particular two-copy NAHR duplication for in-depth discussion and illustration above.

Our model was developed only for detecting NAHR in specific regions predetermined to be susceptible to NAHR. In a sense, our model is orthogonal to existing structural variation (SV) detection algorithms, which detect SVs across the genome but do not handle repeats (and therefore NAHR) well. Thus, while other methods report many more SVs, those they report are from a different class. As in Table 1, 1/3 of our NAHR deletion calls are novel with respect to a comprehensive study by Mills et al. of structural variation encompassing 19 detection algorithms [4]. Overall, 89.8*%* of our NAHR calls are novel, demonstrating that our model detects a different class of SVs (that is NAHR) compared to othermethods.

### Impact on the understanding of non-allelic homologous recombination

#### No hotspots

Since 109 out of 324 distinct NAHR loci experienced an NAHR deletion or duplication event in some individual, then approximately 33.6% of all tested potential NAHR loci were found to be active. Further, among the 109 loci with a positive NAHR event call, the median number of individuals with an NAHR event call at any locus is 5 (11.4%) (see Table 1). This suggests that NAHR activity is fairly common and dispersed across the human genome; it is not concentrated in a small fraction of hotspots out of all potential NAHR loci. The distribution of positive NAHR calls across individuals and loci can be seen in Additional file 1: Figures S9 and S6.

#### Frequency of non-allelic homologous recombination

Our conservative discoveries suggest that NAHR occurs at a much higher frequency than some contemporary estimates in the literature. Studying a pair of related genomic disorders that can arise from NAHR, Liu et al. found the rate of NAHR deletions and duplications during male meiosis to be approximately 10^−5^ to 10^−7^ [54]. Turner et al. developed sperm-based assays to measure the *de novo* rate of NAHR deletions and duplications at four NAHR hotspots in the human genome, also finding the meiotic rates to be approximately 10^−5^ to 10^−7^ [43]. If we assume that the *de novo* rate of NAHR is 10^−5^ genome-wide, then for the 324 potential deletion/duplication NAHR loci we analyzed, we expect 10^−5^×324=0.00324 total *de novo* deletions and duplications per generation. If the difference between an individual and the reference could be quantified as a number of generations, then we would expect 0.324 (0.1*%*) NAHR deletions and duplications to be present in an individual who is separated from the reference by 100 generations. Our analysis found that the median number of NAHR deletion/duplication calls passing the fdr threshold is 24 (7.41%) per individual, much higher than previously thought.

We suspect that this discrepancy with other studies arises because these other studies focus on a few specific disease-related hotspots, while our approach was genome wide. Liu et al. were concerned with a particular region of chromosome 17 in which structural variation can cause serious genetic disorders. Such a sensitive region may not be representative of NAHR rates genome-wide; indeed, it may be more highly conserved due to the demonstrated severe consequences of mutation. Similarly, Turner et al. studied only four NAHR hotspots, and all of them were associated with severe genetic disorders. Thus, the current experimentally estimated rates of NAHR are derived from quite a small sample of regions wherein NAHR causes severe genetic disorders, and thus not necessarily representative of the frequency of NAHR in general.

#### Features correlated with occurrence of non-allelic homologous recombination

A number of genomic architectural features have been hypothesized to play a role in the occurrence of NAHR in the human genome, including LCR length, distance between LCRs, percentage identity of LCRs, distance to telomere or centromere, length of the minimal efficient processing segment (MEPS) [55], distance of breakpoint to MEPS, and several breakpoint motifs. Some studies have empirically calculated the correlation coefficients between the rate of NAHR and some of these features. We tested each of these features with a *χ*^2^ goodness-of-fit test to see if any of them were over- or under-represented in our reliable call set compared to the space of potential NAHR loci we examined. Additional file 1: Table S5 contains the results. The inter-LCR distance and the ratio of the length of LCR to inter-LCR distance were the only features significantly correlated with the occurrence of NAHR (*P*<10^−7^ and *P*<10^−12^, respectively). Details of the various calculations can be found in Additional file 1: Section S7.19.

### Caveats and limitations

Because the conclusions of Bayesian inference are a mathematical consequence of the model and the data, limitations in this work arise from limitations of our model to faithfully represent the underlying biological processes, and from the adequacy of our model of Illumina sequencing technology.

- Our model relies heavily on the assembled human reference genome to define the space of potential NAHR events and loci, and annotate VPs. Thus, our model and subsequent results are impacted by reference bias and assembly errors. For instance, the presence of unassembled LCRs will skew our read-depth likelihood model (the expected read depthswill be too low). Also, unassembled or unannotated LCRs that are in fact hybrid LCRs resulting from recent NAHR events may provide a misleadingset of hybrid reads that are falsely interpreted by our model as evidence of an NAHR breakpoint in an assembled region involving their paralogs.
- Our model also relies heavily on the HSDD to annotate all LCRs. That is, the segmental duplication discovery pipeline used by Bailey et al. to create the HSDD [15] may not annotate all LCRs, and is also susceptible to assembly errors in the reference. Indeed, the presence of unannotated, unassembled, or incorrectly assembled LCRs will affect our analysis of NAHR involving their paralogs. This is especially relevant to our analysis of ancestry, as the referenceis European.
- As described in Section ‘Mechanism of non-allelic homologous recombination’, the gene conversion mechanism is almost identical to the NAHR (crossover) mechanism, and results in hybrid LCRs with breakpoints that mimic those resulting from NAHR. Thus, when we encounter paired-end reads that strongly support a particular breakpoint, it is important to determine if it is an NAHR breakpoint or a gene conversion breakpoint. This decision is further complicated by the fact that, as has been experimentally observed and validated in [3,43] and mentioned in [38], the breakpoint regions of NAHR and gene conversion events may switch between the two mediating LCRs before finally crossing over (NAHR) or not crossing over (gene conversion). Because gene conversion inherently involvespairs of breakpoints, it imposes a severe computational burden on our model. Our inference of gene conversion was therefore limited becausewe considered (for computational concerns) only asmall number of possible breakpoints for eachgene conversion event. As a result, our power to distinguish NAHR from gene conversion was hindered and complicated.
  Indeed, identifying hybrid breakpoint signals as the product of gene conversion or NAHR is especially important for inversions. While we did model inversions, we do not report any inversion results here due to large numbers false positives; presumably gene conversion events mistakenly called as NAHR inversions. Restricting to a set of final calls according to an fdr threshold therefore served a secondary purpose: filtering out false-positive NAHR deletion and duplication calls due to gene conversion complications. In future work, we plan to implement a more sophisticated model for the breakpoint region to more clearly distinguish gene conversion events from NAHR events. Restricting the space of potential gene conversion events among this algorithm’s findings as plausible gene conversion should facilitate their identification.
  Although gene conversion breakpoints are very similar to NAHR breakpoints, our study focused on NAHR deletions and duplications, in which case the change in read depth associated with NAHR deletions and duplications permitted us to correctly interpret hybrid reads as containing evidence of a gene conversion breakpoint (no change in read depth) versus an NAHR deletion or duplication breakpoint (change in read depth).
- All of our positively called NAHR events have an associated called breakpoint, but our confidence in this called breakpoint may vary. When VPs are sparsely distributed across the LCR, it may be impossible (no read can span two VPs) or difficult (few reads can span two VPs) to confidently choose one breakpoint out of many candidates. Also, as noted above, complicated switching has been noted in experimental studies [3,38,43], in which case several different candidate breakpoints may have hybrid read support. In the former case, we can still call an NAHR deletion or duplication event, but there will be insufficient support for the breakpoint to pass the log-odds threshold of Section ‘Breakpoint log odds’. In the latter case, the very notion of breakpoint must be redefined in light of the experimental observations of complicated switching. A more appropriate definition should include the region of complicated switching. This study used the classic notion of a pointwise breakpoint. Expanding our model to allow for complicated switching and redefining the whole notion of a breakpoint is left to future studies.
- Naturally our model has stronger power to detect NAHR events that affect longer stretches of the genome simply due to the larger amount of data available in such cases. See Additional file 1: Section S7.23 for a discussion of the power of our model to detect NAHR.
- Due to computational complexity, we restricted our analysis to a subset (324) of potential NAHR loci among all 1,769 potential NAHR loci involving ≤250 kb implied by the HSDD. More informed conclusions about the features of the NAHR mechanism (see Section ‘Impact on the understanding of NAHR’) could be drawn if we could feasibly analyze a larger set of potential NAHR loci.
- The fdr test we applied is more conservative for deletions relative to duplications: observed-to-expected read-depth ratios are necessarily bounded below by 0, but unbounded above, yet our empirical null distributions were nearly symmetric (see Additional file 1: Section S7.18).
- For computational feasibility, we assumed a simple breakpoint model, where NAHR events have a single switch in VP patterns. Experimental studies show that sometimes the breakpoint region is more complex, with multiple switches in breakpoint patterns [3,43]. Thus, when we call a breakpoint, it may be the case that we have found one of the switches, or one of the breakpoints.
- We perform exact Bayesian inference using our model. This imposes a substantial computational burden (see Additional file 1: Section S7.26). The computational time of our model for analyzing the 324 potential NAHR events for a single low-coverage genome from the 1000 Genomes Project ranged from 1 to 7 days, depending on the number of reads and their average fragment size.
  Using one of the many approximate inference methods instead of exact inference or making appropriate approximations to our model is an attractive option for expanding the number of potential NAHR events across the human genome that are computationally tractable.

## Conclusions

Our specific probabilistic model of NAHR fills an important gap in contemporary analysis of structural variation, and provides a new, biology-inspired computational approach that is nearly orthogonal to existing algorithms. Studies routinely exclude repetitive regions from analysis due to computational and experimental difficulties, and ignore so-called concordantly mapped paired-end reads that contain crucial information of NAHR. As such, our model addresses largely unstudied (from thecomputational perspective) regions of the genome. Nonetheless, repetitive regions and corresponding NAHR rearrangements play important but still mysterious roles in a range of genomic disorders [7,9,10,12,14]. In our analysis, a median of 52 genes were affected by NAHR deletions or duplications per individual. Over the 44 individuals studied here, 216 distinct genes were affected by NAHR events that we called. As long-read DNA sequencing technologies mature, it will be interesting to validate these computational predictions. Moreover, it will also to be useful to combine the advantages of long- and short-read data in improved computational approaches. Improved sequencing technologies coupled with novel computational approaches will help fill in the gaps in our knowledge of NAHR in the human genome.

## Materials and methods

Recall from ‘Results’ that we consider a set of *n*=324 potential NAHR events *E*_1_,…,*E*_*n*_ with associated potential breakpoints *B*_1_,…,*B*_*n*_. Here, *E*_*i*_ represents the type of rearrangement (for example NAHR deletion, duplication and so on) and *B*_*i*_ takes values in the set of VPs between the two mediating LCRs. The data are represented by *D*, which consists of some number *C* of paired-end reads, each of whose nucleotide sequences are denoted by *R* and the generating location by *L*, given as a duple (*R*,*L*). Thus $D = (R,L)_{1}^{C}$.

We calculate the posterior probability of NAHR events and breakpoints via Bayes’ rule:

(1) $$  \mathbb{P} \left((E,B)_{1}^{n} | D \right) = \frac{\mathbb{P} \left(D, (E,B)_{1}^{n} \right) }{ \mathbb{P} \left(D \right)}  $$

that is posterior = joint/marginal. Here, We further specify the joint distribution as:

(2) $$ {\small{\begin{aligned}  \mathbb{P} \left(D, (E,B)_{1}^{n} \right) &= \mathbb{P} \left((R,\cdot)_{1}^{C}, (E,B)_{1}^{n} \right) = \end{aligned}}}  $$

(3) $$ {\small{\begin{aligned} &{\kern2.8pc} \left[ \prod\limits_{i=1}^{C} \sum\limits_{\ell | (E,B)_{1}^{n}} \mathbb{P} \left(R_{i} | \ell, (E,B)_{1}^{n} \right) \!\times \mathbb{P} \left(\ell | (E,B)_{1}^{n} \right) \right]\\& {\kern2.8pc}\times \mathbb{P} \left(C | (E,B)_{1}^{n} \right) \times \left[ \prod_{j=1}^{n} \mathbb{P} \left(B_{j} | E_{j} \right) \right] \times \mathbb{P} \left({E_{1}^{n}} \right). \end{aligned}}}  $$

$\mathbb {P} \left ((R,\cdot)_{1}^{C}, (E,B)_{1}^{n} \right)$ represents the joint distribution of the individual’s genome, which has suspected NAHR events and breakpoints $(E,B)_{1}^{n}$ with respect to the reference, and the data as obtained from the sequencing machine: some number *C* of observed read sequences *R* whose generating locations *L* are hidden (represented by a dot, hence written as (*R*,·)). $\mathbb {P} \left (R_{i} | \ell, (E,B)_{1}^{n} \right)$ gives the likelihood that read *R*_*i*_ was generated from genome position *ℓ*, according to our context-sensitive hidden Markov model read aligner mentioned above. The probability of a read error was conservatively set to 2*%*, with gap-open and gap-extension probabilities of 1*%* and 2*%*, respectively. Read-error probabilities were elevated inside the problematic contexts highlighted in [56-61] and according to cycle, base quality, and homopolymer content. Similarly, gap probabilities were elevated in homopolymers. $\mathbb {P} \left (\ell | (E,B)_{1}^{n} \right)$ is the *a priori* probability of generating location *ℓ* for a given read, where the space of possible mapping locations is affected by the NAHR events and breakpoints $(E,B)_{1}^{n}$, as shown in Additional file 1: Figure S12, and *ℓ* has a uniform distribution over this space. $\mathbb {P} \left (C | (E,B)_{1}^{n} \right)$ evaluates the observed read depth of the NAHR regions given the expected read depth according to the rearrangements $(E,B)_{1}^{n}$. Since read depth has been shown to have larger variation that provided by a Poisson distribution [46], we used a negative binomial, approximated by a normal in large regions. We chose the standard deviation to be 1.05*%* of the mean, following manual inspection of several cases. $\mathbb {P} \left (B_{j} | E_{j} \right)$ is the *a priori* probability of breakpoint *b*_*j*_, which has a uniform distribution over the possible breakpoints for event *E*_*j*_; and $\mathbb {P} \left (e_{j} \right) $ is the *a priori* probability that event *E*_*j*_ occurs, which is the same for all events and has $\mathbb {P} \left (e_{j} = 0 \right) = 1 - 10^{-6}$.

For clarity, we explain Equation 2 in plain English, from right to left. First, we take the reference genome and apply NAHR events ${e_{1}^{n}}$ with corresponding breakpoints ${b_{1}^{n}}$, which has some *a priori* small probability (NAHR events are thought to be rather unlikely), and obtain a hypothetical genome for the individual in question that is completely specified. Then we count the number of reads mapped to various equivalence classes of homology and mapped to unique regions affected by the NAHR events, and calculate the probability of those read-depth counts compared to what should be expected given that $(E,B)_{1}^{n}$ occurred and deleted or duplicated certain sequences in those equivalence classes. Next, we turn to the probability of each read being generated from a given location. Each read is independent, so we take a product across reads. But for each read, we do not know which region generated it, but rather a set of homologous regions, all of which the read maps to reasonably well. The set of homologous regions changes according to $(E,B)_{1}^{n}$. Thus, for each read, rather than using the likelihood of any single mapping location, we instead take the average likelihood across all potential mapping locations given $(E,B)_{1}^{n}$. Finally, we compare all of the hypothetical genomes via Bayes’ rule in Equation 1.

### Availability

The algorithm described in this study, detect-NAHR, is freely available at [37].

## Endnotes

^a^ Pindel actually requires that one mate be mapped uniquely and the other mate to be unmapped. This idea is sufficiently similar to discordantly mapped reads for inclusion here.

^b^ That is, we ranked the low-coverage individuals by the size of their bam file (in gigabytes), and then selected the 44 individuals with the largest bam file.

^c^ We considered a gene to be affected by a specific NAHR event if the gene intersects either of the mediating LCRs or the sequence in between, that is the gene is inside the potential NAHR locus at which the NAHR event was called.

^d^ In reference genome coordinates, *v*_1_ represents chr 1:155184576 and chr 1:155205203, and *v*_2_ represents chr 1:155184704 and chr 1:155205331. Each is a pair of positions because the mediating LCRs are homologous, and mismatches in the pairwise alignment of the mediating LCRs are considered VPs.

^e^ This is theoretically the smallest possible region in which a breakpoint can be called for such an NAHR event. Since there is no other VP between *v*_1_ and *v*_2_, then the sequence spanning [*v*_1_+1,*v*_2_−1] on LCR *A* is identical to the corresponding sequence on LCR *B*. Hence, all NAHR duplications whose breakpoint lies somewhere in [*v*_1_+1,*v*_2_] will have identical resulting hybrid LCRs.

## Additional file

Additional file 1
**Supplementary information [** 55 **,** 62 **-** 82 **].**

## Abbreviations

AHR: Allelic homologous recombination
bp: base pair
DSB: Double-stranded break
fdr: false discovery rate
HSDD: Human Segmental Duplication Database
kb: kilobase
LCR: Low-copy repeat
NAHR: Non-allelic homologous recombination
SNP: Single nucleotide polymorphism
VP: Variational position
