# Supplementary material for: Detecting non-allelic homologous recombination from high-throughput sequencing data
Source: Genome Biol. 2015 Apr 8;16(1):72. doi: 10.1186/s13059-015-0633-1 (PMC4425883; doi:10.1186/s13059-015-0633-1)
Supplement: Additional file 1 — Supplementary information [ 55 , 62 - 82 ]. [file 13059_2015_633_MOESM1_ESM.zip › 13059_2015_633_MOESM1_ESM.pdf]

1 Additional file 1: Supplementary Information

1.1 Additional file 1: Calls per genome

Additional file 1: Figure 6 shows the distribution of number of calls per individual.

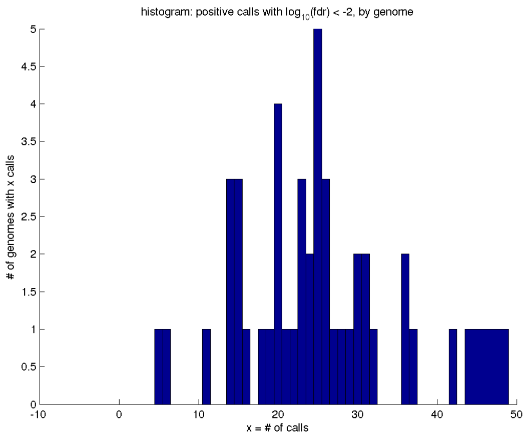

Figure 6 Histogram of the number of positive NAHR calls appearing in an individual.

1.2 Additional file 1: Genes affected by NAHR events

Please see supplementary file `Additional file 2`.

1.3 Additional file 1: Features of LCRs and the rate of NAHR

Please see Additional file 1: Table 3.

feature	p-value	mean theoretical	mean significant	change w.r.t. theoretical mean
LCR length	0.654053	8872.42	6528.66	-26%
inter-LCR distance	**1.83706e-08**	51896.59	17523.47	-66%
LCR length over inter-LCR distance	**8.75584e-13**	0.52	0.87	+66%
log 1 minus percent identity	0.0165497	-3.30	-3.51	+6.5%
distance from telomere	0.0859108	69248964.17	64900872.21	-6.3%
distance from centromere	0.596767	1721505473.66	1829686573.29	+6.3%

Table 3 Features of LCRs and the rate of NAHR. Using the 109 distinct positive NAHR loci, we performed a χ^2 goodness-of-fit test for several features of LCRs that other studies reported to be correlated with rates of NAHR. Only the distributions of inter-LCR distance and ratio of LCR length to inter-LCR distance were found to be statistically significantly different for the 109 positively called NAHR loci compared to the background distribution composed of all 324 potential NAHR loci.

1.4 Additional file 1: Discordant read support for NAHR calls

Please see Additional file 1: Figure 7.

1.5 Additional file 1: Hybrid read support for high-confidence breakpoints

We present a more intuitive summary of breakpoint support in Additional file 1: Figure 8 to supplement the more rigorous probabilistic calculations that gave the breakpoint log-odds. For each of the 512

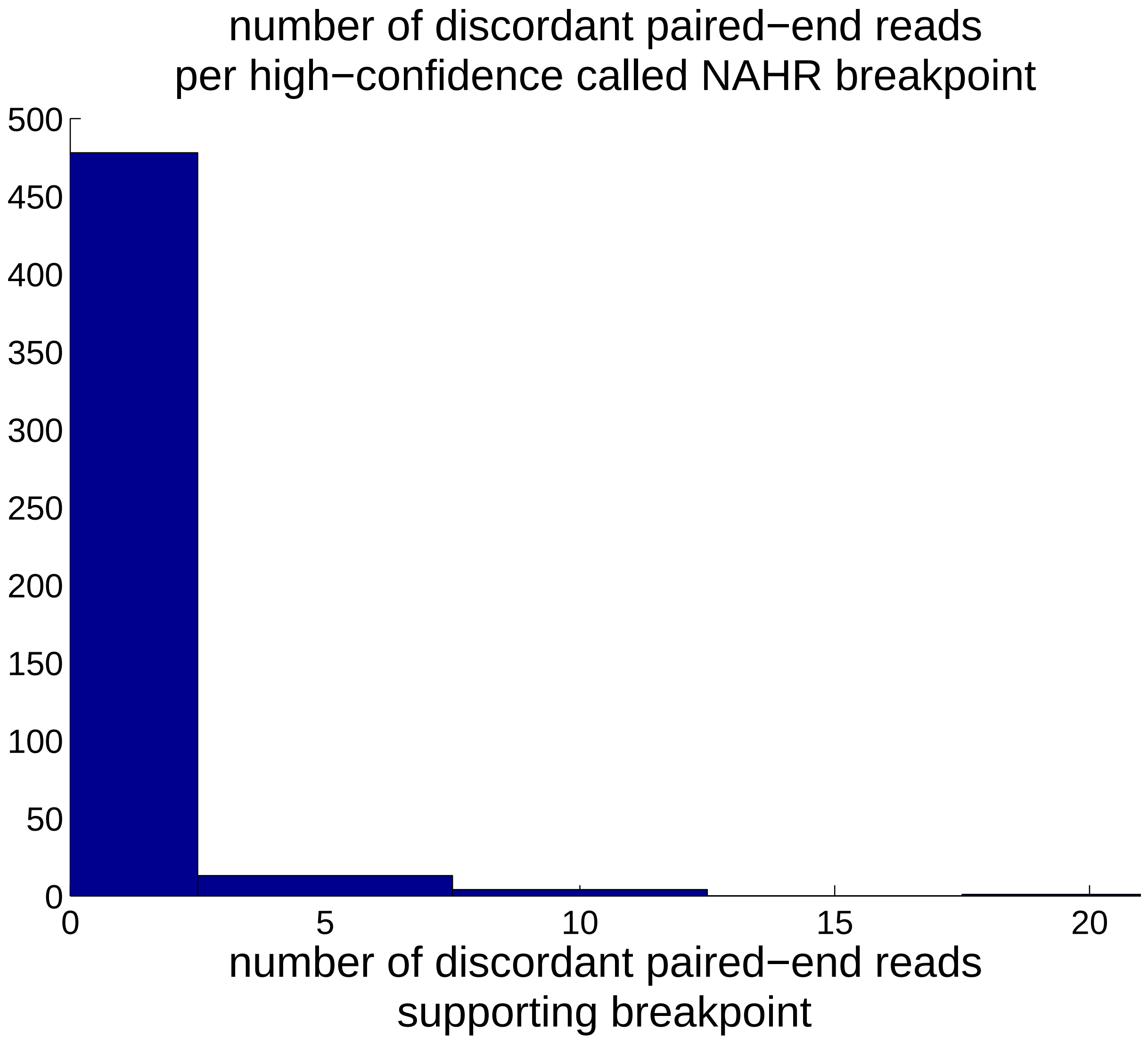

Figure 7 Histogram of the number of discordant paired-end reads supporting high-confidence NAHR breakpoint calls. In particular, 425 (83%) of the 512 NAHR event calls with a high-confidence breakpoint were supported by *zero* discordant paired-end reads, and instead supported only by reads originally mapped with phantom concordance. Further, 478 (93.4%) were supported by ≤ 2 discordant paired-end reads. Since most other structural variation detection algorithms ignore phantom concordant reads and *only* use discordant reads to find structural variation breakpoints, then 425 (83%) of our high-confidence NAHR breakpoint calls are guaranteed to go undetected by other algorithms *by definition*, and another 10.4% would be extremely unlikely to be detected by other algorithms since so few (≤ 2)) discordant reads support them.

NAHR event calls with a high-confidence breakpoint (i.e. breakpoint log-odds ≥ 6), we counted the number of paired-end reads which had a greater probability of being generated from the breakpoint region of the novel hybrid LCR formed by the called NAHR event as opposed to anywhere else in the reference genome.[g] These are the very paired-end reads which display the “switch” in variational position patterns, i.e. the hybrid reads.

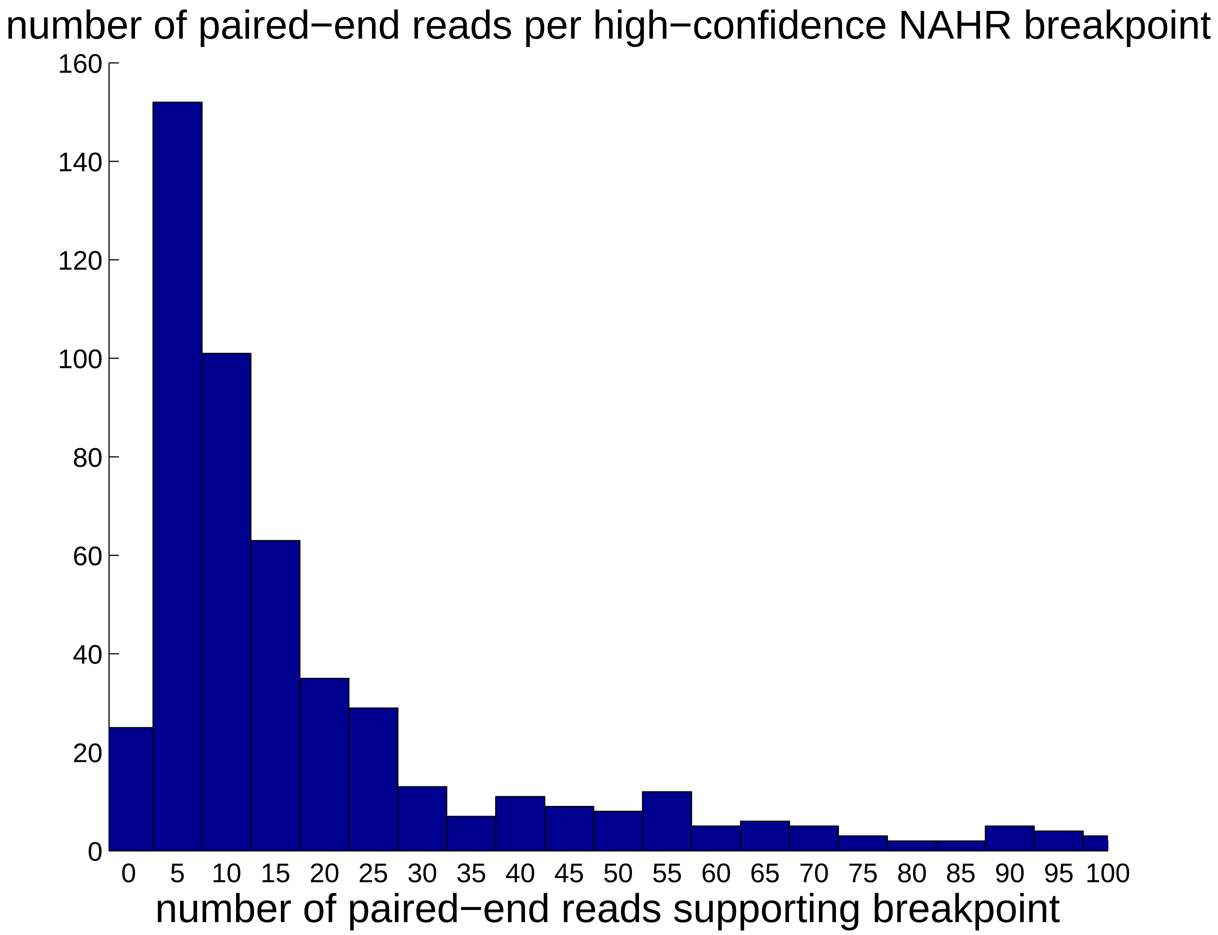

Figure 8 Histogram of the number of paired-end reads supporting high-confidence NAHR breakpoint calls. In total, 512 NAHR event calls had a high-confidence breakpoint. 12 outliers with ≥ 103 paired-end reads not shown. While we computed on low-coverage genomes, unexpectedly large numbers of supporting hybrid reads may be explained by multiple-copy duplications at a single locus or reference sequence errors among homologous LCRs.

1.6 Additional file 1: Previously called rearrangements

We checked our NAHR calls against experimentally validated structural variations reported in three previous studies: Mills, et. al. 2011; Turner et. al. 2008; and Kidd et. al 2010 [3,4,43]. See `Additional file 3` for a list of our calls matched with calls from these previous studies.

1.7 Additional file 1: Separation of positive and negative NAHR event calls by read-depth *fdr* test

Additional file 1: Figure 10 addresses the concern regarding the separation between positive and negative read-depth signals, as one might argue that there is a region of high ambiguity between positive and negative read-depth signals, in which case our *fdr* threshold (0.01) is just an arbitrary cutoff that does not delineate any separation in signal. Of the 109 distinct loci with a positive NAHR event call in some individual, we selected the 31 loci for which between 9 and 35 individuals contained a positive NAHR event call of some kind at the given locus and compared their *fdr* values. The criteria that an NAHR event was called at the same locus in 9 to 35 of the individuals was used so that both sets of log *fdr* values, for positive calls and for negative calls, were nontrivial. For each of these 31 loci, we formed a boxplot of the log *fdr* values for those individuals with a positively called NAHR event at the locus and a boxplot of the

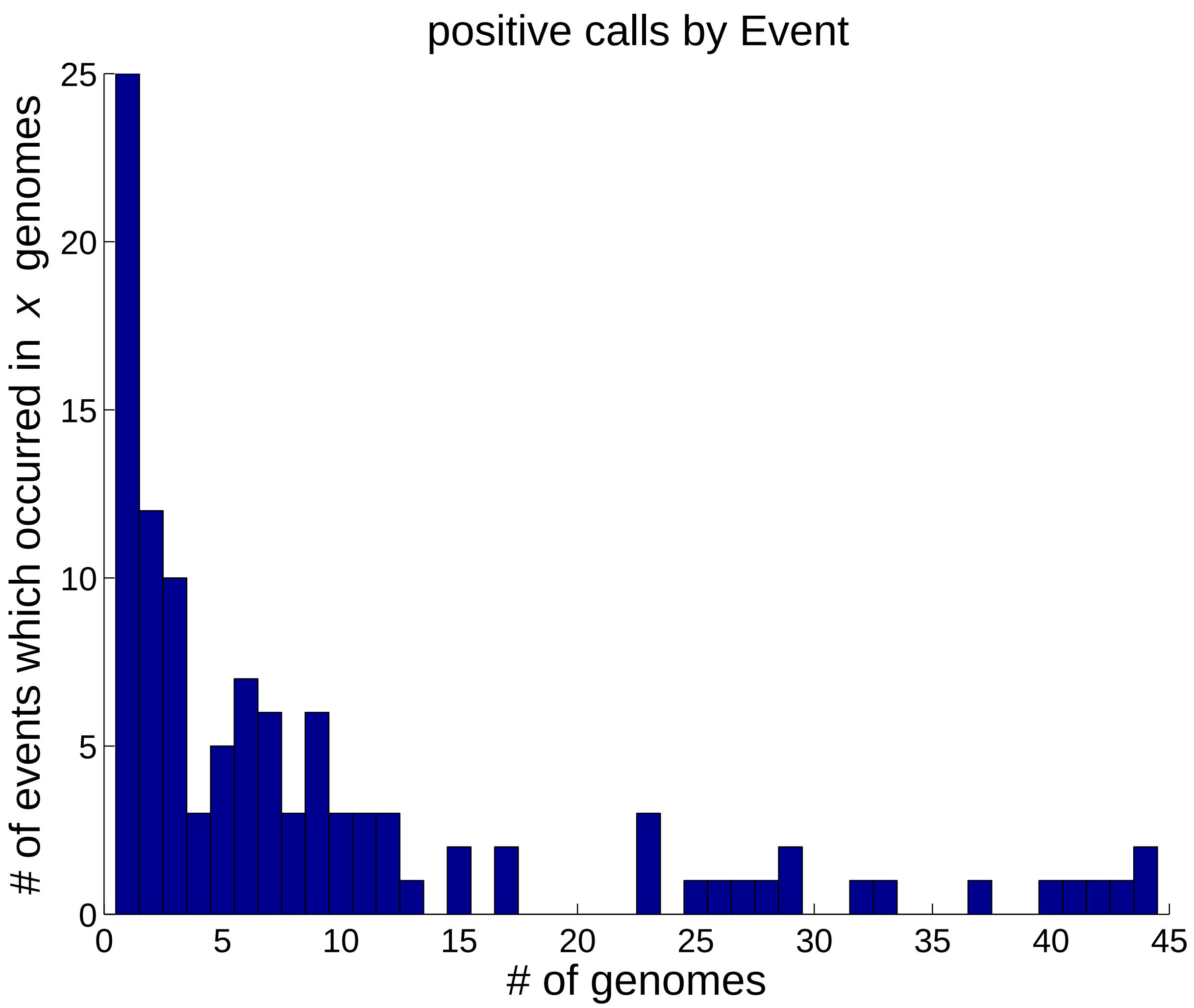

Figure 9 Distribution of positive NAHR event calls by number of individuals. Note that out of 109 distinct loci with a positive NAHR event call, only 7 (6.4%) loci were called as positive in ≥ 34 (77.3%) of the tested individuals. Among loci with a positive NAHR event call, the median number of individuals with a positive call at any particular locus was 5 (11.4%).

log *fdr* values for those individuals with a negative NAHR call. It is clear from Additional file 1: Figure 10 that these two populations are very well separated among the 31 loci. Notice that, in Additional file 1: Figure 10, whenever there is an occasional overlap in whiskers, it is always the case that the negative calls' log *fdr* extends into the very low *fdr* region. These are presumably merely a small number of false negatives. To illustrate the signal difference with a specific example, we selected potential NAHR locus chr 19 : [8336858, 8366563]. We called a homozygous deletion for Japanese individual NA18973 at this locus, and we called no NAHR deletions or duplications for Yoruban individual NA18523 at this locus. These calls are credible from looking at the read-depth graphs alone. Further, NA18973 has $fdr = 1.2 \times 10^{-3}$ and NA18523 has $fdr > 0.99$.

1.8 Additional file 1: Other important examples of NAHR

Please see Additional file 1: Table 4.

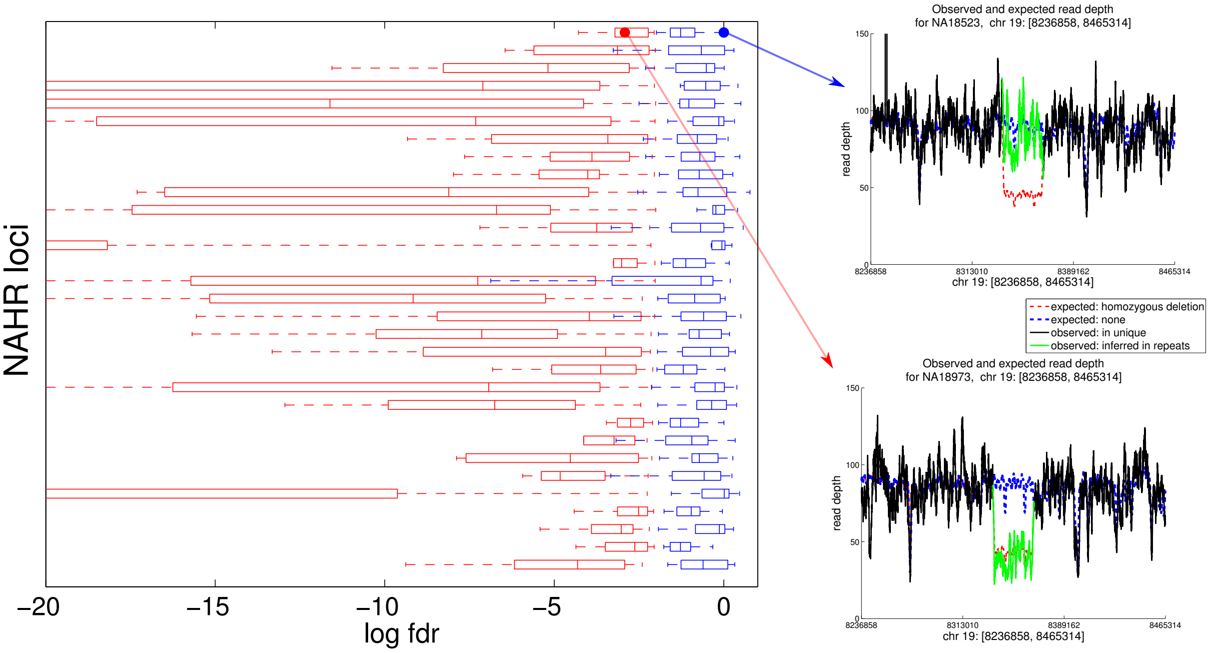

Figure 10 Boxplots of the $\log fdr$ **for** 31 **loci with a positive NAHR event call.** Of the 109 distinct loci with a positive NAHR event call in some individual, we selected the 31 loci for which between 9 and 35 (out of 44) individuals had a positive NAHR event call at the locus. For each of the 31 loci, we created a boxplot (red) of $\log fdr$ for those individuals with a positive event call at the locus, and a separate $\log fdr$ boxplot (blue) for those individuals with a negative event call at the locus. Within a locus, the positive and negative boxplots are adjacent. Whisker lengths cover up observations within up to 1.5 of the interquartile range. For simplicity, outliers are not shown. For the potential NAHR locus chr 19 : [8336858, 8366563], we plot the read-depth for Japanese individual NA18973 for whom we called a homozygous deletion at this locus, and Yoruban individual NA18523 for whom we did not call an NAHR deletion or duplication at this locus. Read-depth inferred in repeats is shown in green, observed read-depth in unique regions is shown in black, expected read-depth given no NAHR deletions or duplications is shown in blue, and the expected read-depth given a homozygous NAHR deletion is shown in red. Read-depth is plotted as 1250 bp-wide moving sums. NA18973 has $fdr = 1.2 \times 10^{-3}$ and NA18523 has $fdr > 0.99$.

1.9 Additional file 1: Gene context of NAHR duplication at chr1 : 155, 184, 704 – 155, 205, 331 for NA19129 (case study)

Please see Additional file 1: Figure 11.

1.10 Additional file 1: Hybrid read alignments for negative examples NA07051 and NA18501

Please see `Additional files 4 and 5`.

1.11 Additional file 1: Reference genome

We used hg19 / GRCh37 as the reference genome.

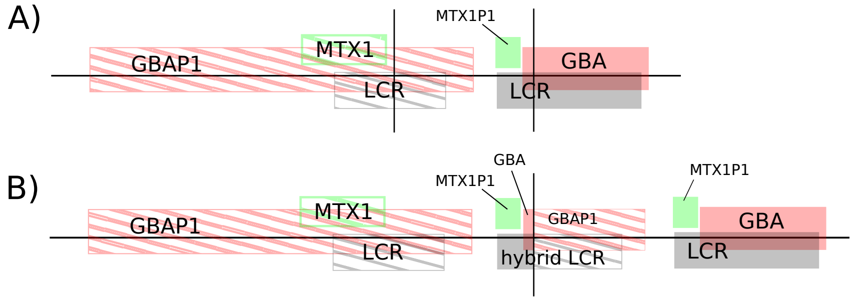

Figure 11 Effect of the called NAHR duplication in NA19129 on the *GBA* gene. A) Gene context of the locus of the studied NAHR two-copy duplication as it appears in the reference genome (i.e. before the called NAHR two-copy duplication). The mediating LCRs are gray. The first LCR is a 10.6 kbp segment of the $GBAP1$ pseudogene and contains the latter 3.5 kbp of $MTX1$. The second LCR consists of pseudogene $MTX1P1$ and all but the last 1.4 kbp of *GBA*. The breakpoints of the called NAHR duplication are vertical black lines. **B)** The same locus, after the called NAHR duplication. The hybrid LCR formed from the duplication contains an extra copy of pseudogene $MTX1P1$ and a novel fusion gene, consisting of the first 1.1 kbp of *GBA* followed by the last 12.5 kbp of pseudogene $GBAP1$. The breakpoint region (containing the switch in variational positions) is marked by the vertical black line.

1.12 Additional file 1: The Human Segmental Duplication Database and homology maps

The Human Segmental Duplication Database (HSDD) lists pairs of genome intervals that are $\geq$ 1kb in length and $\geq$ 90% sequence identity [15]. It was obtained from http://humanparalogy.gs.washington.edu. The LCRs listed in the HSDD range in length form 1 kb to 770 kb. The HSDD does not list the variational positions for any pair of LCRs - we inferred these from the pairwise alignment of all homologous pairs of LCRs listed in the HSDD. The distribution of variational positions varies considerably within a pair of LCRs and between different pairs.

Performing a pairwise alignment of the sequences associated with each pair of genome intervals, we obtain a set of maps $m_1, \ldots, m_n$ between homologous regions of the genome. For a given position x in the reference genome, we define the positions homologous to x as $\mathcal{M}(x) := \{x\} \cup \{z : \exists i \text{ s.t. } m_i(x) = z \text{ or } m_i(z) = x\}$. Then unique positions in the genome (as far as the HSDD is concerned) have $\mathcal{M}(x) = \{x\}$.

1.13 Additional file 1: Relationships between LCRs

Please see Additional file 1: Figure 12.

1.14 Additional file 1: Context-sensitive conditional HMM

Many studies have analyzed the error rates and biases of base generation in Illumina reads, showing that error rates in Illumina machines are context-specific, increase with cycle, increase with homopolymer length, and are not adequately quantified by associated quality scores [55-60]. We have designed a context-sensitive conditional hidden Markov model read aligner which accounts for these biases and error-rates, using conservative estimates for the probability of a correct base and exaggerated estimates of biases and error-rates, to calculate the probability that a paired-end read was generated from a given location.

Our context-sensitive conditional hidden Markov model has the structure of the conventional HMM for pairwise sequence alignment: the states are `match`, `insertion`, and `deletion`; and emissions are nucleotides `A`,`C`,`G`,`T`. However, contrary to the conventional pairwise HMM, all emission and transition

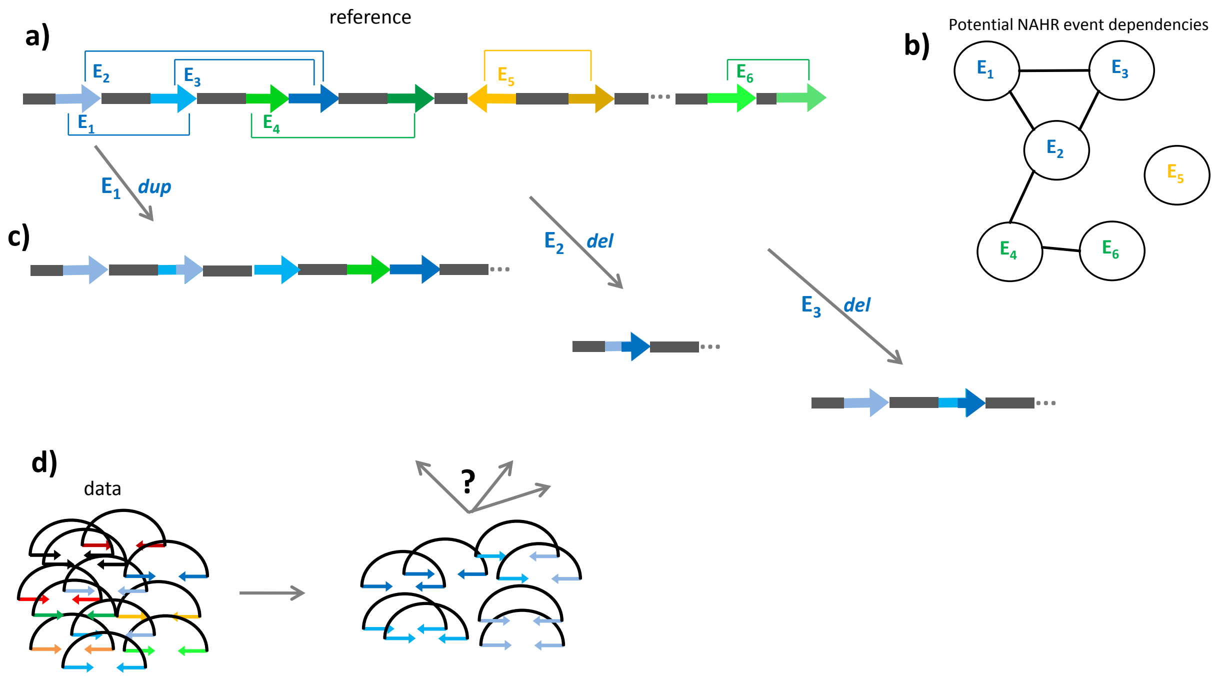

Figure 12 Schematic example of the relationship between LCRs, NAHR events, and high-throughput sequencing data. **(a)** At the top is a hypothetical chromosome. Homologous regions are different shades of the same color, dark gray regions are unique regions, and arrow direction marks orientation of the LCRs. Various potential NAHR events are labeled $E_1, \ldots, E_6$, and their mediating LCRs are indicated by brackets. **(b)** Several separate hypothetical NAHR events are shown: a duplication at E_1, a deletion at E_2, and a deletion at E_3. Each event shown results in a distinct, new hybrid LCR that was not in the original genome. Notice that these three events are related by *exclusivity constraints*: only one of E_1, E_2, E_3 may occur at a time. **(c)** From the hypothetical group of all paired-end reads, we identify the subset which are homologous to blue regions. We would then align this subset of paired-end reads to the LCRs resulting from the different hypothetical NAHR event scenarios to obtain their likelihoods (not shown). Among the subset of "blue" data, notice the paired-end reads with one light blue mate and one medium blue mate. These would suggest the presence of the hybrid medium blue-light blue LCR that results from the E_1 duplication. **(d)** A graph representing the dependencies between the potential NAHR events in (a). NAHR events at separate potential NAHR loci must be evaluated simultaneously if the intervals affected by the events overlap or share homology. Here, E_2, E_3, and E_4 all have overlapping potentially affected regions, and hence E_2, E_3, E_4 are related by *exclusivity constraints*, represented by a dotted line. On the other hand, the regions affected by E_1 and E_4 are not overlapping but are homologous (they both affect "blue" LCRs), and hence E_1 and E_4 must be considered simultaneously, but they do not have an exclusivity constraint.

probabilities are *conditional* on the reference sequence. That is, when aligning sequences X and Y, where X is a read and Y is a substring of the reference, then the emission and transition probabilities are parameterized according to certain sequence features of Y.

The parameters were determined as follows. The per-position base error rate was conservatively set to 2%, i.e. at every position in every read, the probability of an error was at least 2%. This error-rate was then increased for positions along the read that satisfied certain criteria, described below. These criteria were drawn from both confirmed and suspected sources of bias and error mentioned in several studies of Illumina error-rates and biases [55-60]. In the following, a context written as $A_1A_2A_3$ means that $A_1A_2A_3$ are three consecutive bases in the reference genome, and that $A_)$ and A_2 were previously read by the machine, and the machine is currently reading A_3. Also, $\mathtt{X}$ represents any nucleotide, i.e. $X \in \{\mathtt{A,C,G,T}\}$.

- `GGX` context: add 1% error.
- `AGX` context: add 1% error.
- `GX` or `TX` context: add 1% error.
- Let L be the length of the read. Then add 1% error if the position being read is in $[\frac{1}{4}L, \frac{1}{2}L)$; add 2% error is the position is in $[\frac{1}{2}L, \frac{3}{4}L)$; and add 3% error is the position is in $[\frac{3}{4}L, L]$.
- Let A_i be the position currently being read, and suppose $A_i = A_{i-1} = A_{i-2} = \cdots = A_{i-m} \neq A_{i-(m+1)}$ for some m. If $m \in [2, 5]$, add 2% error. If $m \geq 6$, add 4% error.
- If $A_1A_2A_3$ is the context, and $A_1 = A_2 \neq A_3$, then add 1% error.
- If the position being read is one of the first 4 bases of the read, add 1% error.
- A Phred quality score Q is given for each position in a read, where $Q := -10 \cdot \log_{10}(P_{err})$ where P_{err} is an estimate of the error probability for the position being read (this is generated by the machine). We could then add $10^{\frac{Q}{-10}}$ to our error-rate. To be extra conservative, we instead add $10^{\frac{Q \cdot 0.80}{-10}}$, i.e. we artificially *worsen* the quality.

If a position satisfies multiple of the above situations, we add all of the relevant error rate penalties. Note that all of these values are strongly conservative: we have increased the error-rate for sources of bias described in the literature that are both tested and untested (i.e. merely suspected), and both our base error rate and the additional error rate penalties are much higher than those suggested in the aforementioned relevant studies.

Suppose that adjusting the error-rate according to the above penalties results in proposed error-rate p'_e. To get the real error-rate, we simply cap this at 73%, i.e. $p_e = \min(p'_e, 0.73$. Then the probability of reading the correct base is simply $1 - p_e$, and the probability of any other base is $\frac{p_e}{3}$.

We follow a similar approach for transitions probabilities. We set the probability of transition to match = 98%, to an insertion or deletion from match = 0.5%, and extending and insertion or deletion = 2%. If inside of a homopolymer of length ≥ 3, i.e. $A_i = A_{i-1} = A_{i-2} = \cdots = A_{i-m}$ with $m \geq 2$, then we change the above transition probabilities to 94%, 3$, and 5%, respectively.

1.15 Additional file 1: GC-bias and the per-position fragmentation rate

We follow Benjamini & Speed 2012 [46] in the construction of GC-bias sensitive per-position fragmentation rate along the reference genome. Benjamini & Speed concluded that the GC-bias affecting paired-end read distribution along the genome is correlated with the number of G's and C's in the *fragment* which produced the paired-end reads.

Let F be the nucleotide sequence of the reference genome. Define the function $c_\mu(x) := \sum_{y \in [x, x+\mu-1]} \mathbf{1}_{F_y \in \{G,C\}}$, i.e. $c_\mu(x)$ counts the number of G's and C's in the hypothetical fragment of length μ starting at position x in the reference genome. Let $N_\mu(z) := \left|\{x : c_\mu(x) = z\}\right|$, i.e. the number of positions in the reference genome with GC-count z in the fragment of length μ starting at that position.

Consider a paired-end read library $\mathcal{L}$ with average fragment length μ whose reads have been mapped to the reference genome. For simplicity, we will ignore the actually observed fragment length implied by a given mapped paired-end read. Instead, for a paired-end read whose leftmost aligned position in the reference genome is position x, we consider the observed fragment to be the interval $[x, x + \mu - 1]$ in the reference. Let

$$O_{\mathcal{L}}(z) := \text{number of observed fragments in } \mathcal{L} \text{ with GC-count } z. \tag{6.0.1}$$

Finally, we define the fragmentation rate for library $\mathcal{L}$ with average fragment length μ at position x in the genome as $\lambda_{\mathcal{L}}(x) := \frac{O_{\mathcal{L}}(c_\mu(x))}{N_\mu(c_\mu(x))}$. If there are multiple paired-end read libraries $\mathcal{L}_1, \ldots, \mathcal{L}_n$, then we calculate the overall fragmentation rate $\lambda(x) := \sum_{i=1}^{n} \lambda_{\mathcal{L}_i}(x)$.

1.16 Additional file 1: Pseudo-mapping probability within repeats

Consider a paired-end read R whose left-endpoint is position x with $\mathcal{M}(x) \neq \{x\}$, i.e. the read maps to a repetitive region. For each $z \in \mathcal{M}(x)$, the small (fragment-sized) region L_z about z represents a paralogous mapping location for R. *A priori*, R is equally likely to have come from each L_z, and so $\forall z \in \mathcal{M}(x), \mathbb{P}(L_z) = \frac{1}{|\mathcal{M}(x)|}$. We align R to each paralog L_z to obtain $P_z := \mathbb{P}(R \big| L_z)$, i.e. the probability that R was generated from paralog L_z. Then we form the posterior distribution on paralogs

$$\mathbb{P}(L_z \big| R) := \frac{\mathbb{P}(R \big| L_z) \cdot \mathbb{P}(L_z)}{\sum\limits_{w \in \mathcal{M}(x)} \mathbb{P}(R \big| L_w) \cdot \mathbb{P}(L_w)} \tag{6.0.1}$$

$$= \frac{\mathbb{P}(R \big| L_z)}{\sum\limits_{w \in \mathcal{M}(x)} \mathbb{P}(R \big| L_w)} \tag{6.0.2}$$

Finally, suppose X is a repetitive region. Let $R_1, \ldots, R_n$ be the set of all paired-end reads which are homologous to X, i.e. $\forall i$, if M_i is the set of possible mapping location for read R_i, then $M_i \cap X \neq \emptyset$. We calculate γ for the region X as

$$\gamma = \frac{\sum_{i=1}^{n} \sum_{z \in M_i \cap X} \mathbb{P}\left(L_z \big| R_i\right)}{\sum_{x \in X} \lambda(x)} \tag{6.0.3}$$

1.17 Additional file 1: Details of the *fdr* test

We wish to develop a statistical test for our calls based on read-depth alone which did not mimic the read-depth part of our full model. For a statistic, we choose to use the ratio γ of observed read-depth to GC-sensitive expected read-depth under the null (no event). The expected number of reads is calculated according to the GC-sensitive fragmentation rate, as described in Additional file 1: Section 1.15. Thus, the chosen statistic γ does not depend on the length of the region and is intended to account for GC-content bias.

Following Efron 2004, we view the false discovery rate from the Bayesian perspective: the observed empirical distribution f of the test statistic γ is really a mixture of two distributions: f_0, the distribution of γ under the null hypothesis, and f_1 the distribution of γ under the alternative. We could then calculate the *fdr* (actually, an upper-bound for the FDR) as

$$fdr(\gamma) := \frac{f_0(\gamma)}{f(\gamma)} \tag{6.0.1}$$

The important question is then: what is the distribution of γ under the null hypothesis? We would naturally suppose that $\gamma \sim N(1, \sigma^2)$ for some σ^2. But this assumes that our GC-sensitive read-depth model is quite good, which is not necessarily true. Substantial progress has been made in identifying biases in coverage in Illumina sequencing machines. While Benjamini & Speed 2012 showed the bias to be related to the GC content of potential fragments [46], not all sources of bias have been investigated, and no complete, verified model with all parameters learned is available.

Efron developed the above perspective of *fdr* in the context of normalized z-values for some test. In such a scenario, the z-values for data coming from the alternative hypothesis are thought to be generally far from 0, while the z-values for the data generated from the null hypothesis are thought to be generally close to 0. The distribution f_0 of the z-values coming from the null hypothesis in Efron's case would traditionally be assumed to be $N(0, 1)$. But Efron shows that if f_0 differs even slightly from $N(0, 1)$, then it has important implications for the consequent FDR calculations and thus for identifying a multiple-comparison corrected statistically significant subset of the data. Efron proposed that when a large amount of data is available, f_0 can instead be estimated empirically from a subset of the data. Therefore, Efron suggested that if <∼ 10% of the data is believed to come from the alternative hypothesis, then the distribution of the z-values under the null can be estimated using only the data around the large, central peak near 0, as most datapoints producing z-values in this region near 0 are assumed to be from the null [48].

We adapt Efron's approach and apply a similar idea to our case for the ratio γ. First, we fit $f(\gamma)$ from the data of all potential NAHR loci across all individuals; $44 \times 324 = 14,256$ datapoints. For regions that did not experience an NAHR deletion or duplication, we expect γ to generally be near 1. For regions that did experience an NAHR deletion or duplication, we expect γ to be much less than 1 (for deletions) or much greater than 1 (for duplications). Like Efron, we estimate the location and scale of the distribution obtained from the control regions to the central peak of f, which is near 1. We have a considerable advantage in our case: an expansive control region of the genome unlikely to have experienced an NAHR deletion or duplication. Therefore, for each individual, we can estimate the distribution f_0 under the null using data from the "control region" of the genome: the unique regions, away from LCRs and potential NAHR loci, where we may safely assume that there are relatively very few genome rearrangements.

For a given individual, let the observed distribution of γ among the potential NAHR loci be f. As in Efron 2004 [48], f can be obtained by smoothing the observed empirical distribution of all γ (we used lowess smoothing). Thus, to calculate the *fdr* as in Additional file 1: Section 6.0.1, we only need an expression for f_0. Below, we detail how to construct the distribution f_0 of γ under the null distribution, and how to calculate γ in unique and repeat regions.

1.17.1 Constructing f_0

For each individual, and for each potential NAHR locus tested, we randomly selected a contiguous region of the same length as the potential NAHR locus from the "control region" of the reference genome (away from LCRs and potential NAHR loci, not near the centromere or telomere) and calculated its observed-to-expected ratio γ. For each individual, we thus empirically estimated $f_0^c :=$ the distribution of γ under the null hypothesis in the control region of the genome.

It turns out that the means of the empirically estimated f_0^c for each individual vary considerably, as shown in Additional file 1: Figure 13. They also considerably deviated from Normal (QQ plots not shown). This serves as confirmation that the GC-sensitive coverage model learned from the literature is somewhat problematic. We also concluded that 1) the *fdr* for each individual should be calculated separately, i.e. *not* by pooling observations across individuals, 2) we should *not* assume that γ has mean

1 under the null hypothesis for each individual, and instead 3) fit a separate 2-component Gaussian mixture for f_0^c to each individual, denoted as

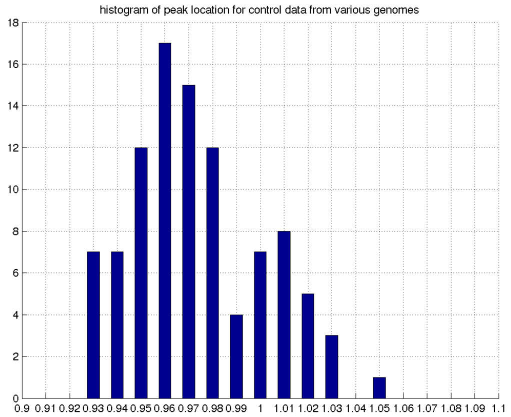

Figure 13 Histogram of the central peak location for the observed-to-expected read-depth ratios for data from the control regions of the 44 individuals tested. Notice the considerable variation in central peak location - this suggests that we should *not* simply assume that the observed-to-expected read-depth ratio γ has mean 1. Indeed, it seems that the GC-sensitive fragmentation rate calculated according to the literature actually overestimates the expected read-depth.

$$f_0^c(\gamma) := p_0 \cdot g(\gamma; \mu_0, \sigma_0^2) + p_1 \cdot g(\gamma; \mu_1, \sigma_1^2), \tag{6.0.2}$$

where g is the Gaussian density.

We observed that the central peaks of f_0^c and the observed distribution f for potential NAHR loci differed considerably. Thus, to derive a distribution for γ for potential NAHR loci leveraging the information from the control region, we enforced a series of constraints to transform f_0^c into an appropriate f_0.

For a given individual, let the observed distribution of γ in the potential NAHR loci be f. As in Efron 2004, f can be obtained by smoothing (we used lowess smoothing). To estimate f_0, Efron suggested using the center and half-width of the central peak in f to define a Normal distribution. In this spirit, we transform f_0^c into f_0 by minimizing the Euclidean distance between their half-widths and central peaks, as follows.

For some function h, let $P_c^h := (x_c^h, y_c^h)$ be the geometric location of the central peak of h, i.e. $h(x_c) = y_c$ and $y_c = \text{argmax}_y\, h(y)$. Let $P_\ell^h := (x_\ell^h, y_\ell^h)$ be the geometric location of the lower half-width, i.e. $h(x_\ell^h) = y_\ell^h = \frac{y_c^h}{2}$ with $x_\ell^h < x_c^h$. Similarly, $P_u^h := (x_u^h, y_u^h)$ be the geometric location of the upper half-width, i.e. $h(x_u^h) = y_u^h = \frac{y_c^h}{2}$ with $x_u^h > x_c^h$. Also, let $d(P_1, P_2)$ be the Euclidean distance between points P_1, P_2. We define an adjustment to the control Gaussian mixture in Additional file 1: Equation 6.0.2 as

$$f_{\alpha,\beta}(\gamma) := p_0 \cdot g\Big(\gamma; \mu_0 + \alpha, (\sigma_0 \cdot \beta)^2\Big) + p_1 \cdot g\Big(\gamma; \mu_1 + \alpha, (\sigma_1 \cdot \beta)^2\Big), \tag{6.0.3}$$

where, again, g is the Gaussian density.

Finally, we set $f_0 := f_{\alpha^*,\beta^*}$ where

$$(\alpha^*,\beta^*) = \underset{(\alpha,\beta)}{\operatorname{argmin}}\, d(P_\ell^f, P_\ell^{f_{\alpha,\beta}}) + d(P_c^f, P_c^{f_{\alpha,\beta}}) + d(P_u^f, P_u^{f_{\alpha,\beta}}) \tag{6.0.4}$$

1.17.2 Calculating γ in unique regions

In the unique regions of the NAHR loci, there is no ambiguity in the mapping of reads: we can safely assume that the read really was generated from the location it was mapped to with probability = 1. Thus, for unique region U, we calculate γ as

$$\gamma = \frac{\textit{\# of paired-end reads with left-endpoint } \in U}{\sum_{x \in U} \lambda(x)} \tag{6.0.5}$$

using the notation for λ from Additional file 1: Section 1.15.

1.17.3 Calculating γ in repeat regions

This is the same procedure as in Additional file 1: section 1.16.

1.18 Additional file 1: Empirical null distributions for FDR tests

As described in Additional file 1: Section 1.17, for each of the 44 individuals tested, we empirically estimated a separate distribution f_0 of the observed-to-null-expected read-depth ratio γ under the null hypothesis. Each f_0 was obtained by fitting a 2-component Gaussian mixture to control data and then translating both means by α and scaling both standard deviations by β. The mixture coefficients p_i, means μ_i, and standard deviations σ_i, for $i = 0, 1$, and the adjustment values α, β for each individual are in `Additional file 6`.

1.19 Additional file 1: Calculation of NAHR features

The features of interest are as follows:

Length of LCRs NAHR events are mediated by a pair of homologous LCRs, which are likely not the same length due to indels, but close. For a potential NAHR event, we calculate the length of its LCRs as the length of the pairwise alignment of the two mediating LCRs.

Distance between LCRs Following Liu et. al., 2011, we calculate the inter-LCR distance as the "length of the segment in between LCRs plus the length of one LCR" [54].

LCR length over inter-LCR distance This follows directly from the above two definitions.

Sequence identity For a potential NAHR event, we performed a pairwise alignment of its two mediating LCRs. Every position in the alignment with a mismatch or that is part of a small indel was considered a variational position. The percent divergence was calculated as $t = \frac{\text{number variational positions}}{\text{length of alignment}}$. We used $\log t$ to measure the association between sequence identity and occurrence of NAHR.

Proximity to centromere/telomere We calculated the distance to the centromere as the distance from the closest index out of both mediating LCRs to the closest index to the centromere, i.e. $\left| \max(\text{centromere coordinates}) - \min(\text{both LCR coordinates}) \right|$. The distance to the telomere was calculated analogously, using the telomere on the same chromosome arm as the two mediating LCRs.
For each feature above, we calculated the distribution f of the feature using all 324 distinct loci tested, and the observed distribution f of the feature only for those 109 distinct loci with an NAHR deletion or duplication call in at least one individual. We then performed a χ^2 goodness-of-fit test to determine if the difference between f and g was statistically significant. The results are shown below.

Length of LCRs Several studies have hypothesized a relationship between the length of the mediating LCRs and the rate of NAHR [11,12,38,74]. Liu et. al. 2011 [54] found a positive correlation between NAHR rate and LCR length. We did *not* find the length of mediating LCRs to be statistically significantly associated with the occurrence of NAHR (p-value = 0.65).

Distance between LCRs The distance between LCRs has also been thought to play a role in the rate of NAHR [11,12,38,74]. Liu et. al. 2011 [54] found a negative correlation between NAHR rate and inter-LCR distance, as hypothesized. We found that the distance between the mediating LCRs was statistically significantly shorter than expected (p-value = 3.9×10^{-9}, mean background inter-LCR distance = 52 kbp, mean observed inter-LCR distance = 17 kbp). This result is additionally believable since our model has stronger power to detect NAHR events that affect longer stretches of the genome.

LCR length over inter-LCR distance Liu et. al. 2011 [54] found the strongest correlation to be between rate of NAHR and the ratio of LCR length over inter-LCR distance. Our results agree: we found the ratio to be statistically significantly higher among occurring NAHR loci compared to background (p-value = 7.75×10^{-13}).

Sequence identity The overall degree of sequence identity between mediating LCRs has also been thought to play a role as well [74]. We found that sequence identity is statistically significantly higher among occurring NAHR events compared to background (p-value = 0.0108).

Proximity to centromere/telomere The subtelomeric and pericentromeric regions of chromosome have been noted to be hotspots of recombination and enriched with segmental duplications [11,75,76]. We tested whether NAHR occurred in these regions at a rate higher than would be expected due to the layout of LCRs across the genome. We found that NAHR is statistically significantly over-represented near the telomere (p-value = 0.041), but does not deviate significantly from the background for proximity to the centromere.

1.20 Additional file 1: Simulation procedure

1. randomly draw 20 NAHR events and breakpoints;
2. apply the drawn NAHR events and breakpoints to the reference to obtain a modified reference genome;
3. simulate $15X$ paired-end read data from the diploid genome consisting of one copy of the modified reference genome and one copy of the reference genome;
4. run our model on the simulated data.

Each step above is described in greater detail below:

1. The 20 NAHR events were randomly drawn from the space of 324 potential NAHR events that were analyzed on real data, without replacement. The draws respected the exclusivity constraints (rules of NAHR depicted in Additional file 1: Figure 12). The NAHR breakpoint was drawn as follows. We drew a position v from a uniform distribution on the length of the pairwise alignment profile of the two mediating LCRs. The drawn NAHR breakpoint was declared to be $\text{argmin}_{v_i > v}\, v_i$, i.e. the first variational position after v. In this way, the sampling of the NAHR breakpoint respected the distance between neighboring potential breakpoints.

   All draws were considered to be one-copy (heterozygous) rearrangements. The full diploid simulated genome was considered to be one copy of the reference genome (hg19), and one copy of the rearranged genome described here.

2. This is straightforward, following the mechanics of NAHR.

3. Paired-end read data was simulated using pIRS [77] with the following parameters: `-i` $< modified_g enome >$ `-I` $< hg19_r eference_g enome >$ `-l 76 -x 15 -m 300 -Q 33 -g 0`

   In words, these options specify: reads have length 76bp, average fragment size is 300, and coverage is $15X$. Note that pIRS approximates distribution in read errors and read quality.

4. This is straightforward.

1.21 Additional file 1: Aligning reads to hybrid LCRs

We illustrate the process of constructing NAHR breakpoint regions in a hypothetical genome, which are novel with respect to the reference genome, and aligning relevant paired-end reads to them. We use an NAHR deletion as an example.

Consider two homologous LCRs X and Y on the same chromosome arm and with the same orientation. Let E be the random variable representing an NAHR event between X and Y, and let B be the corresponding breakpoint. Suppose we are considering the case in which E results in an NAHR deletion with breakpoint $B = v_i$, where v_i is the ith variational position in the profile of X and Y.

Read data is given in the form of a `.bam` file of paired-end reads aligned to the reference genome via some aligner like BWA [50]. Since the NAHR event $E = deletion, B = v_i$ results in a hybrid LCR XY that is novel with respect to the reference genome, then for none of the paired-end reads in our dataset (i.e. `.bam` file) do we have an alignment to the novel hybrid LCR XY. Now, we easily identify all of the paired-end reads which *might* have been generated from the hybrid LCR XY - they are simply any paired-end read which has been mappd to LCR X or Y or any paralog of X or Y. The set of paralogoous regions is obtained from the HSDD (see section Additional file 1: 1.12).

Now, of all the paired-end reads homologous to XY, we can easily identify the subset which may have been generated from the NAHR breakpoint junction, i.e. potential hybrid reads, as follows. Consider the small neighborhood V about v_i, e.g. $V = [v_i - d, v_i + d]$ where d is the average fragment length plus several standard deviations. We collect all paired-end reads $r_1, \ldots, r_m$ from any region in X, Y, or their paralogs which is homologous to V, where the homology is determined via the homology maps described in section Additional file 1: 1.12.

We then construct the proposed NAHR breakpoint region at v_i exactly as follows. Recall that we are considering an NAHR deletion between X and Y at v_i. Let $x_{(i)}$ the be position on X corresponding to v_i, and $y_{(i)}$ the analogous on Y (i.e. in the pairwise alignment of X and Y, $x_{(i)}$) and $y_i(i)$ are matched

together and their index in the alignment profile is v_i). According to the rules of NAHR, the sequence of the hybrid LCR XY resulting from an NAHR deletion between X and Y with breakpoint v_i is given by $x_1 x_2 \ldots x_{(i)-1} y_{(i)} x_{(i)+1} \cdots y_n$.

We then use the context-sensitive conditional HMM (Additional file 1: section 1.14) to perform pairwise alignments between each relevant read $R_1, \ldots, R_m$ and the hybrid LCR XY given above. In fact, since the reads $r_1, \ldots, r_m$ came from a neighborhood of radius d about v_i, then the pairwise alignments are performed against only a neighborhood of radius d about the breakpoint in XY (slightly larger than d, to be safe).

1.22 Additional file 1: Variational positions vs. paralogous sequence variants

We describe here the distinction between a *variational position* (VP) and a *paralogous sequence variant* (PSV).

Suppose LCRs $X, Y,$ and Z are the same length and are paralogs. Suppose that at position w, X and Y have nucleotide `A` and Z has nucleotide `C`. Then position w is a PSV for $X, Y,$ and Z. But if we consider the potential NAHR event mediated by X and Y, then position w does not vary between X and Y. Thus, w cannot be a breakpoint between X and Y, as we defined breakpoint to be a position at which X and Y vary. Thus, w is a PSV for X, Y, Z, but w is *not* a variational position between X and Y.

1.23 Additional file 1: Power to detect NAHR

The power of our model to detect NAHR events depends on several factors. Detecting the *locus* of an NAHR deletion or duplication event depends on the size of the locus and the depth of coverage. Since our definition of NAHR requires the mediating LCRs to have length $\geq$ 1 kb, then any potential NAHR locus has size $\geq$ 2 kb. Thus, in principle, with any reasonable depth of coverage, there is always power to detect the *locus* of an NAHR deletion or duplication event because of the change in read-depth signal.

On the other hand, the power to detect the precise breakpoint within a locus depends on the *distribution* of variational positions along the mediating LCRs - *not* necessarily the number of VPs. This is rather nuanced and even counter-intuitive. For example, detecting an NAHR deletion or duplication event between 100% identical tandem repeats is easy: the read-depth signal is strong, and the breakpoint is trivial (any position will do since they are identical!). For a more complicated example, consider two VPs v_1, v_2 where $v_2 = v_1 + 100$, and suppose the reads have fragment length 300 and mate length 75. Even though v_1, v_2 are relatively closely spaced with respect to fragment length, an NAHR breakpoint at or v_2 will be undetectable, since no paired-end read with such fragment length and read length properties can overlap v_1 and v_2 within the same mate, or have one mate overlapping v_1 and the other overlapping v_2. If there is a third VP $v_3 = v_1 + 250$, then an NAHR breakpoint at v_2 or v_3 could be detected but not distinguished between v_2 and v_3.

In summary, the power detect NAHR deletion or duplication events is quite good overall due to the minimum size of a potential NAHR locus, while the power detect an NAHR breakpoint is data- and breakpoint-specific rather than locus-specific - it depends on the relative locations of the VPs surrounding the breakpoint and the fragment length and read length of the paired-end reads.

1.24 Additional file 1: Justification of the use of the HSDD and the criteria for defining a potential NAHR event

As noted in several experimental studies, frequency of NAHR appears to be positively correlated with repeat length and degree of homology, and negatively correlated with the distance between the repeats

[6,11,54]. This observation supports our criteria for the definition of a potential NAHR event - that the mediating LCRs be ≥ 1000 bp in length and ≥ 90% identity.

Further, studies of the fundamental step in homologous recombination mechanisms - the 5′ single-stranded DNA homology search process - suggest that quite a long stretch of near perfect homology is necessary to initiate homologous recombination. That is, immediately following a double stranded break (DSB), the exposed 5′ strand on both sides of the DSB is resected, leaving dangling 3′ single-stranded DNA (ssDNA) tails [78]. Mediated by the Rad51 protein, one of the 3′ ssDNA tails searches for highly homologous double-stranded DNA (dsDNA). Consecutive nucleotides in prospective dsDNA are analyzed in triplets [79], and dsDNA is rejected if sequence divergence becomes unacceptably high [80]. The criteria for selecting a sufficiently homologous sequence are stringent: recombination rate drops off exponentially as sequence identity decreases from 100% [81]. The length requirement over which high homology must be present, known as the Minimal Efficient Processing Segment (MEPS) [82], is believed to be in the range of 300 − 500bp for humans [8,11].

Since such a long stretch (300 − 500 bp) of nearly identical sequence is necessary to initiate homologous recombination, we believe that the Human Segmental Duplication Database (HSDD), which contains all pairs of LCRs with ≥ 90% identity and ≥ 1000 bp length, is a reasonable foundation for constructing the space of potential NAHR events in the human genome. Afterall, the ssDNA search process plausibly has a much higher probability of finding a suitable minimal efficient processing segment (MEPS) in an LCR of length ≥ 1000 than in an *Alu* of length 500 - since the *Alu* is approximately the length of the required MEPS, then the searching ssDNA would have to find the beginning of the *Alu*, whereas there are presumably many location within a long LCR wherein a suitable 300 − 500 bp MEPS might be found.

1.25 Additional file 1: Breakpoint log-odds threshold

Suppose the probability of a read-error is p. The the numerator and denominator in the log-odds breakpoint ratio are products across reads of sums over mapping locations. Recall that the reads are chosen as the reads which map best to the hybrid than to anywhere else in the genome. Since the error rate p is rather small, we approximate the sum by the largest summand. Then the odds ratio of the breakpoint region to the null is approximately $\left(\frac{1-p}{p}\right)^m$.

We imposed a breakpoint threshold of 6, i.e.

$$m > \frac{6}{\log_{10}[\frac{1-p}{p}]}, \tag{6.0.1}$$

. This can be interpreted as: there are m instances in which a read correctly matches a variational position in the alignment to the hybrid ($1 - p$ in the numerator), but mismatches that same variational position in the alignment to one of the mediating LCRs. These m instances can represent m different alignments, e.g. if there is only one other variational position nearby to the called breakpoint, in which the difference between an alignment to the hybrid and to the original mediating LCRs differs by one position. These m instances can also come from fewer than m alignments, e.g. if there are multiple variational positions in the proximity of the called breakpoint, and a single alignment spans several of these variational positions.

Additional file 1: Equation 6.0.1 depends on the error-rate in read alignments. As described in Additional file 1: section 1.14, our error-rates were quite conservative, and were liberally increased under various conditions believed to be associated with elevated error-rates. Thus, Additional file 1: equation 6.0.1 is an oversimplification, as p varies. Nonetheless, we calculate here Additional file 1: equation

6.0.1 for various values of p to get an idea of the effect on m.

For $p = 0.02$ (our base error-rate), $m = 3.5$; for $p = 0.05, m = 4.7$; for $p = 0.10, m = 6.3$. Thus, the breakpoint threshold requires a *minimum* of somewhere between 4 and 7 instances in which a variational position is correctly matched when the read is aligned to the hybrid, but incorrectly matched at that same variational position when the read is aligned to the mediating LCRs. Again, this could mean 4 – 7 separate reads if the variational positions around the called breakpoint are sparse, or fewer if there are several variational positions in the vicinity of the called breakpoint.

1.26 Additional file 1: Computational burden

1.26.1 Determining computational complexity

Consider a potential NAHR locus A_i, i.e. a pair of homologous LCRs on the same arm of the same chromosome. If the LCRs comprising A_i have the same orientation, then A_i may experience one of the following homologous recombination outcomes: S'_i :=*null,del,dup,gene-conversion X donates to Y, gene-conversion Y donates to X*; on the other hand, if the LCRs comprising A have opposite orientation, then the possible outcomes are: S'_i := *null,inv,gene-conversion X donates to Y, gene-conversion Y donates to X.*

We assume a diploid human reference genome, so there are two copies of A_i - one on each copy of the two copies of the chromosome (recall that we excluded sex chromosomes). The potential NAHR event E_i is then a random variable representing the occurrence or absence of an NAHR (or gene conversion) event at locus A_i on each of the two chromosome copies. Since each chromosome copy is independent of the other, then the sample space of E_i is $S_i := S'_i \times S'_i$.

As per the rules of NAHR and illustrated in Additional file 1: Figure 12, two NAHR events must be considered simultaneously if their loci contain homologous LCRs (either as the mediating LCRs or in the region inbetween the mediating LCRs) or overlap. This defines a graphical model on the space of potential NAHR events E_1^n. In this graphical model, a node is a potential NAHR event E_i, and edges connect two events if their loci share homologous LCRs or overlap.

In this graphical model, the computational complexity of a single node E_i is then $\prod_{j \in \eta(E_i)} S_j$, where $\eta(E_i) = \left\{ j : E_j \text{ is a neighbor of } E_i \right\} \cup \{i\}$. For example, if there are 3 potential NAHR events that form a clique, and the mediating LCRs of each have the same orientation, then the computational complexity of any node in the clique is $(5 \times 5)^3 = 15,625$. Clearly, the computational burden is substantial, due to the diploid nature of our model.

We can improve the computational complexity somewhat by taking note of *exclusivity constraints*, as follows. Consider two *overlapping* potential NAHR event loci A_1, A_2 on chromosome α with corresponding potential NAHR event random variables E_1, E_2 (respectively). Certain outcome configurations are obviously impossible, for example, E_1 and E_2 cannot both be deletions on the same copy of the chromosome α. For example, suppose E_1 results in a NAHR deletion on the first copy of chromosome α. Now, since A_1 and A_2 are overlapping, then one of the mediating LCRs of A_2 has been deleted on the first copy of chromosome α. Thus, an NAHR event cannot occur at A_2 on the first copy of chromosome α, i.e. $E_2 \in null \times S'_2$ with probability $= 1$. We include duplication and inversion outcomes in this exclusivity constraint as well. While the same argument does not hold up as for deletions, extending exclusivity constraints to the case of duplications and inversion is like assuming that multiple NAHR events did not occur on the same copy of the same chromosome at the same loci at different generations. Since NAHR events at a particular genomic location are thought to be rare to begin with ($O(10^{-5})$ to $O(10^{-7})$ per loci per generation [43,54]), we believe extending the exclusivity constraints in this way is

a reasonable approximation.

1.26.2 Criteria for our study

There are 1769 potential NAHR loci/events across the human reference genome (hg19), where potential NAHR loci are defined by the HSDD (Additional file 1: section 1.12). We ran our model on all potential NAHR events in connected components which satisfied the following criteria: 1) the connected component had total computational complexity < 2000; 2) none of the events in the connected component have homology to either sex chromosome (i.e. have homologous LCRs on a sex chromosome); 3) all events in the connected component have loci that are completely defined in the reference genome (some potential NAHR loci had long strings of `N` nucleotides in their sequence, representing unsequenced parts of the reference genome).

Out of 1769 potential NAHR events across the entire reference genome, there are 535 potential NAHR events in connected components with total compute size < 2000. Of these 535 potential NAHR events, 324 of them satisfy the criteria above and have mediating LCRs with the same orientation (i.e. may result in an NAHR deletion or duplication, but not an NAHR inversion) - these are the ones we analyzed. 79 potential NAHR events are in connected components with computational complexity between $2,000$ and $10,000$. The remaining 1150 potential NAHR events lie in connected components with total compute size $> 10,000$.

Because our algorithm is computationally demanding (re-aligning all reads on an LCR to each paralog and hypothetical hybrid LCRs using the context-sensitive conditional HMM), the latter 1229 events cannot be feasibly computed with exact inference. Algorithms for approximate inference on graphical models or approximations to the model are certainly an option for analyzing the more computationally burdensome connected components, but these avenues were not explored in the current study.

The computational time of our model, analyzing all connected components with total compute size < 2000 for a single low-coverage genome from the 1000 Genomes Project, ranged from 1 to 7 days, depending on the number of reads and their average fragment size. The BAM files for the 44 individuals we studied ranged from 20 – 63 GB.

1.27 Additional file 1: Table of calls

Please see `Additional file 7`. Please cite this paper if our list of NAHR predictions serves as motivation for further investigation of NAHR events at any of these loci.

Table 4 Example novel detections

affected genes of interest	molecular functions & associated diseases of selected genes	reference context	resultant genes	call	breakpoint coordinates in reference	read-depth	breakpoint log-odds	individual
RNASE2, RNASE3	RNASE2: ribonuclease; RNASE3: asthma, immune host defenses [61,62]	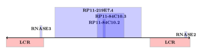	fusion gene: RNASE2→RNASE3	duplication	chr 14: [21360223, 21424308]	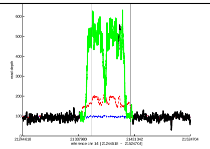	13.2	NA18949
FLG	FLG: Ichthyosis vulgaris, atopic dermatitis, asthma, allergic rhinitis, food allergy [63]	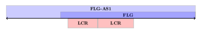	expanded FLG	duplication	chr 1: [152276781, 152281644]	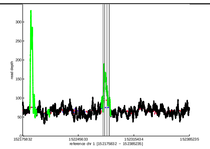	28.2	NA19204
CYP2E1, SPRN, SYCE1	CYP2E1: drug metabolism, diabetes, obesity, fasting, alcohol & non-alcohol liver disease [64]. SPRN: Creutzfeldt-Jakob disease [65]. SYCE1: synaptonemal complex of meiosis [66]	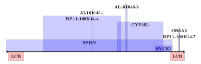	duplicated CYP2E1, SPRN, SYCE1	duplication	chr 10: [135249727, 135393855]	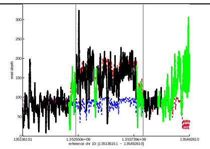	5.5	NA18501
HP, HPR, TXNL4B	HP: haptoglobin, diabetic cardiovascular disease [67], Crohn's Disease [68], & others [69]. HPR: protects against Trypanosomiasis [70]. TXNL4B: ribosome assembly [71], pre-mRNA splicing, interacts with Prp6 [72,73]	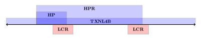	duplicated part of HPR, expanded TXNL4B	duplication	chr 16: [72095319, 72111510]	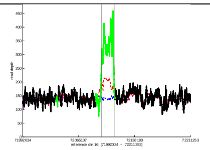	33.9	NA19108
